# Supplementary material for: A socially interdependent choice framework for social influences in healthcare decision-making: a study protocol
Source: BMJ Open. 2024 Mar 8;14(3):e079768. doi: 10.1136/bmjopen-2023-079768 (PMC10928740; doi:10.1136/bmjopen-2023-079768)
Supplement: Author's manuscript [file bmjopen-2023-079768.draft_revisions.pdf]

BMJ Open is committed to open peer review. As part of this commitment we make the peer review history of every article we publish publicly available.

When an article is published we post the peer reviewers' comments and the authors' responses online. We also post the versions of the paper that were used during peer review. These are the versions that the peer review comments apply to.

The versions of the paper that follow are the versions that were submitted during the peer review process. They are not the versions of record or the final published versions. They should not be cited or distributed as the published version of this manuscript.

BMJ Open is an open access journal and the full, final, typeset and author-corrected version of record of the manuscript is available on our site with no access controls, subscription charges or pay-per-view fees (http://bmjopen.bmj.com).

If you have any questions on BMJ Open's open peer review process please email info.bmjopen@bmj.com

BMJ Open

A Social Interdependent Choice Framework for Social Influences in Healthcare Decision-Making: A Study Protocol

Journal:	*BMJ Open*
Manuscript ID	bmjopen-2023-079768
Article Type:	Protocol
Date Submitted by the Author:	12-Sep-2023
Complete List of Authors:	Nouwens, Sven; Erasmus Universiteit Rotterdam Erasmus School of Health Policy and Management; Erasmus University Rotterdam, Erasmus Choice Modeling Centre<br>Veldwijk, Jorien; Erasmus Universiteit Rotterdam Erasmus School of Health Policy and Management; Erasmus University Rotterdam, Erasmus Choice Modeling Centre<br>Pilli, Luis; Erasmus Universiteit Rotterdam Erasmus School of Health Policy and Management; Erasmus University Rotterdam, Erasmus Choice Modeling Centre<br>Swait, J.D.; Erasmus Universiteit Rotterdam Erasmus School of Health Policy and Management; Erasmus University Rotterdam, Erasmus Choice Modeling Centre<br>Coast, Joanna; University of Bristol, Population Health Sciences<br>de Bekker-Grob, Esther; Erasmus Universiteit Rotterdam Erasmus School of Health Policy and Management; Erasmus University Rotterdam, Erasmus Choice Modeling Centre
Keywords:	Social Interaction, HEALTH ECONOMICS, Social Support

SCHOLARONE™
Manuscripts

A Social Interdependent Choice Framework for Social Influences in Healthcare Decision-Making: A Study Protocol

S.P.H. Nouwens[1,2,3*], J. Veldwijk[1,2,3], L. Pilli[1,2,3], J.D. Swait[1,2], J. Coast[4], E.W. de Bekker-Grob[1,2,3]

[1] Erasmus School of Health Policy & Management, Erasmus University Rotterdam

[2] Erasmus Choice Modeling Centre, Erasmus University Rotterdam, the Netherlands

3 Erasmus Centre for Health Economics Rotterdam, Erasmus University, the Netherlands

[4] Health Economics Bristol, Population Health Sciences, Bristol Medical School, University of Bristol, UK

* Correspondence to:

S.P.H. Nouwens

Erasmus School of Health Policy & Management

Erasmus University Rotterdam

P.O. Box 1738

3000 DR Rotterdam

The Netherlands

nouwens@eshpm.eur.nl

For peer review only

Funding Statement

The project has received funding from the Dutch Research Council (NWO-Talent-Scheme-Vidi-Grant No. 09150171910002). Joanna Coast is supported by the Wellcome Trust [205384/Z/16/Z]. For the purpose of open access, the author has applied a CC BY public copyright license to any Author Accepted Manuscript version arising from this submission.

Competing Statement

All authors have completed the ICMJE uniform disclosure form at http://www.icmje.org/disclosure-of-interest/ and declare: no support from any organisation for the submitted work; no financial relationships with any organisations that might have an

Classification: Internal

interest in the submitted work in the previous three years; no other relationships or activities that could appear to have influenced the submitted work.

Word Count: 3405

Keywords: Social Interaction, Social Environment, Decision-Making, Patient Preferences, Choice Behaviour

For peer review only

Classification: Internal

Abstract

Objectives Current choice models in healthcare (and beyond) can provide suboptimal predictions of healthcare users' decisions. One reason for such inaccuracy is that standard microeconomic theory assumes that decisions of healthcare users are made in a social vacuum. Healthcare choices, however, can in fact be (entirely) socially determined. To achieve more accurate choice predictions within healthcare and therefore better policy-decisions, the social influences that affect healthcare user decision-making need to be identified and explicitly integrated into choice models. The purpose of this study is to develop a social interdependent choice framework of healthcare user decision-making.

Design A mixed methods approach will be used. A systematic literature review will be conducted that identifies the social influences on healthcare user decision-making. Based on the outcomes of a systematic literature review, an interview guide will be developed that assesses which, and how, social influences affect healthcare user decision-making in four different medical fields. This guide will be used during two exploratory focus groups to assess the engagement of participants and clarity of questions and probes. The refined interview guide will be used to conduct the semi-structured interviews with healthcare professionals and users. These interviews will explore in detail which, and how, social influences affect healthcare user decision-making. Focus group and interview transcripts will be analysed iteratively using a constant comparative approach based upon a mix of inductive and deductive coding. Based on the outcomes a social influence independent choice framework for healthcare user decision-making will be drafted. Finally, the Delphi technique will be employed to achieve consensus about the final version of this choice framework.

Ethics and dissemination This study was approved by the Erasmus School of Health Policy & Management Research Ethics Review Committee (ESHPM, Rotterdam, The Netherlands; reference ETH2122-0666). Findings will be published in peer-reviewed academic journals and further shared with various audiences such as researchers, healthcare professionals and policy makers through methods such as public presentations and academic conferences.

Strengths and weaknesses of this study

- This is the first study that will attempt to develop a social interdependent choice framework of healthcare user decision-making.

For peer review only

Classification: Internal

- Through applying a mixed method approach, existing knowledge and empirical evidence will be combined to ensure a rigorous approach for framework development.
- Inclusion of articles based on the study's title and abstract in the systematic literature review step may result in potentially relevant areas remaining unexamined. In part, this will be corrected in the interviews and Delphi method steps, which allow influences and influence mechanisms to surface later in the study.
- Participant recruitment via healthcare providers may lead to selection bias. However, recruited participants will likely be experienced with healthcare decision-making within a medical field of interest.

For peer review only

Classification: Internal

Introduction

Rising expenditures, an ageing population, high prices of new medical treatment options, and substantial annual resource waste put healthcare under heavy pressure (1-4). To avoid poor policy decisions, trial-and-error implementation and demand-supply imbalance, there is a need for accurate predictions of healthcare users' choice behaviour (5, 6). Current choice models in healthcare (and beyond), however, provide inaccurate predictions of individual healthcare user decisions. One reason for such inaccuracy is that standard microeconomic theory assumes that decision-making occurs in a social vacuum. Choice behaviour, under this *homo economicus* perspective (7), is regarded as a completely rational process in which the decision maker is driven purely by their own interest, disregarding any self- or other-originating psychological factors such as social influences. However, healthcare user choices can in fact be (entirely) socially determined (8-12). To achieve more accurate choice behaviour predictions within healthcare and therefore better policy-decisions, the social influences that affect healthcare users' decision-making need to be identified and explicitly integrated into choice models.

This motivates the need for an expanded choice paradigm that must be developed and validated. In the pursuit of this objective, a first important step is to develop and extend the theory of decision-making by building a conceptual framework that depicts which social influences impact healthcare user decision-making, and how they achieve their impact. With this in mind, we developed a systematic approach that 1) empirically identifies these social influences and 2) suggests mechanisms for their impacts. We strive towards these objectives using a phased approach: a systematic literature review, a series of focus groups and semi-structured interviews, and the Delphi method will supplement each other. This paper provides the protocol for the systematic approach that will be taken towards developing this social interdependent choice framework.

Objectives

This study is part of the INTERSOCIAL project that aims to develop and validate a social interdependent choice paradigm for healthcare user decision-making such that more accurate predictions of choice behaviour in healthcare can be achieved (13). This study protocol will focus on conducting the necessary steps that will guide the attempt to extend the existing theoretical choice framework to account for social influences. The objectives for this study protocol are:

Classification: Internal

1. to identify which, and how, social influences have previously been found to affect healthcare user decision-making based on existing published peer-reviewed studies;
2. to explore with healthcare users in four different areas which, and how, social influences affect healthcare user decision-making; and
3. to develop consensus among experts (including healthcare professionals) about which, and how, social influences affect healthcare user decision-making.

Definition of Social Influence

There exist many definitions of social influence (14-17) with different levels of practical applicability. Based on a consensus meeting with the research team and external experts (n=16) from different academic fields, it was decided to use the following definition in the present study: "*social influence* is the process by which an individual's behaviour, thoughts, beliefs, attitudes, feelings, actions, preferences, decisions, or choice behaviour are (directly and/or indirectly) affected by other people." These 'other people' can be anyone that the decision maker (in our case, users of healthcare) is in direct contact with but also individuals that the decision maker is not in direct contact with, e.g., celebrity endorsements (18).

Note that our definition is centred on a decision maker whose healthcare decisions may depend upon other people in the decision maker's social circle, implying that we are excluding the study of networks of individual healthcare users who are jointly influenced by each other (19). We apply a unidirectional paradigm where the entire social network is centred on a single healthcare user and study how this social network ((types of) individuals and processes) influences the healthcare user, but not how the healthcare user influences others' social networks or how a healthcare user's social contacts are influenced by each other. We only include social influences that can be tracked to a single source and incorporate other social influences (such as culture (20), education (21) and perceived discrimination (22)) as potential covariates only.

Research Design

The research process will use a mixed-methods approach. First, a systematic literature review that identifies the (types of) individuals and the processes that systematically influence healthcare users' decisions within different medical fields will be performed. The results from the systematic literature review will aid in constructing an interview guide for the subsequent round of (group and individual) interviews. As individuals are often unconscious and unaware

For peer review only - http://bmjopen.bmj.com/site/about/guidelines.xhtml

Classification: Internal

of the degree to which they are socially influenced (23, 24), the results from the systematic literature review can additionally help to construct interview questions that stimulate recall and awareness of social influences that might otherwise not be raised by respondents. Second, two focus groups and forty semi-structured interviews will be conducted to explore and assess healthcare users' and healthcare professionals' views on social influences on healthcare user decision-making. The focus groups have a secondary aim of testing engagement of participants with the topic and assessing the clarity of questions and probes in the interview guide. Their feedback will then be used to refine the interview guide for the semi-structured interviews. Third, the Delphi method will be applied to reach consensus among experts (including healthcare professionals and choice modellers) about the social interdependent choice framework on healthcare user decision-making.

For the systematic literature review the following medical fields of study have been chosen: immunization, birth setting, arthroplasty, and prostate cancer treatment. The rationale for choosing these fields is fourfold. First, healthcare users in these fields face a degree of autonomy in their choice, meaning that they are not obliged to choose one alternative over the other, which is essential for testing real choice behaviour predictability. Second, many healthcare users face these medical fields (in the Netherlands, which has 17.5 million inhabitants: almost all people are affected by immunization, between 160,000 and 180,000 people give birth yearly (25), 31,514 hip replacements were reported in 2022 (26), and prevalence of prostate cancer is over 55,000 (27)) speaking to relevance from the perspective of development of an extended social interdependent framework. Third, a higher degree of generalizability is achieved by including medical fields that are likely to differ in types/degrees of social interdependence and consequences that may employ different mechanisms of social influence. Fourth, in each of these four fields, there are current policy challenges related to the decisions made by healthcare users in these areas: i) the demand for influenza vaccination is lower than for other vaccinations (28), ii) there is increasing interest in birth centres in the Dutch population (29), iii) there is an exponential rise in patients with joint problems (30-31), and iv) there is an alarming decrease in active-surveillance compliance from prostate cancer patients (32).

Systematic Literature Review

Initially, a systematic literature review of social influences on healthcare user decision-making will be performed. The review will follow PRISMA guidelines (33) and map the

For peer review only - http://bmjopen.bmj.com/site/about/guidelines.xhtml

Classification: Internal

literature in three dimensions that concern the development of the social interdependent choice paradigm proposed by the INTERSOCIAL project (13). These dimensions describe i) the individuals or groups influencing health-related decision-making; ii) the mechanisms through which they influence decisions of healthcare users; and iii) the choice constructs that these processes affect. A narrative synthesis of the sources and mechanisms of social influence and choice constructs affected will be provided.

Concerning the first dimension, psychosocial variables are one of the key facets of patient-centred care (34) and the past literature offers evidence of treatment choices as an action embedded in a network of social relationships, including experts and non-experts. This dimension informs whose preferences or actions must be considered for the development of the theoretical framework for healthcare user decision-making.

The second dimension, i.e., social influence mechanisms, refers to the processes by which a healthcare user's behaviour, thoughts, beliefs, attitudes, feelings, actions, preferences, decisions, or choice behaviours are (directly and/or indirectly) affected by other people. Mechanisms and dimensions already present in the health-related literature will support classifying included papers into categories. Physician-patient interaction (35, 36) and social support (37) are two relevant and well-defined constructs in the domain of healthcare user decision-making. Social norm is a relevant construct from socio-cognitive theories, and it is present in many attitudinal-behavioural models in the health domain, such as the Health Belief (38) model, I-Change (39, 40) model, and the Transtheoretical Model of Behavior Change (41).

The creation of a social interdependent choice framework demands the connection between the first two dimensions and the choice constructs involved in choice econometric models. Since insights about this connection from the extant literature are sparse at best, choice constructs will be aggregated into three broadly defined main categories. These categories contain the effects of social influences on (1) healthcare users' goals, (2) preference formation, and (3) non-evaluative components of choice models. Goals involve crucial personal trade-offs such as life expectancy versus quality of life, where different alternatives are valued differently based on the goal that a healthcare user seeks to attain vis-à-vis the other persons involved in the decision-making. Preference formation describes the effects of social influences on the acquisition of information about attributes and weights deployed to healthcare users to determine options' total utilities. Finally, non-evaluative components refer

For peer review only

Classification: Internal

to aspects such as behavioural rules, non-compensatory processes, or screening of alternatives before evaluation.

To attain the objectives of the systematic literature review, nine different databases will be searched: Embase, Cochrane CENTRAL, Econlit, Google Scholar, International Bibliography of the Social Sciences, Medline, Psychinfo, Scopus, and Web of Science. To be eligible, a paper must return from a search containing (variations around) the keywords “social influence” AND “decision-making” AND “empirical data” AND “healthcare field”. The “healthcare field” differs for each of the four healthcare fields studied. For example, the field of immunization will use the keywords “vaccine” OR “vaccine hesitancy” OR “vaccination” OR “vaccination coverage”. We will exclude the terms “parental” and “parental decision-making”, as we are interested in how a healthcare user makes choices about their own health and how social influences affect these choices.

The inclusion criteria further require that a paper has the full-text available, is written in English, and presents results of original empirical data collected in Western countries. Countries that will be included in the search strategy are the United States, Canada, North America, United Kingdom, England, Ireland, Northern Ireland, Scotland, The Netherlands, Belgium, France, Germany, Denmark, Norway, Sweden, Finland, Spain, Portugal, Italy, Austria, Switzerland, Iceland, Luxembourg, Liechtenstein, San Marino, Monaco, Andorra, Malta, Cyprus, Gibraltar, Europe, Australia, and New Zealand. Papers reporting data from other countries will be excluded due to the assumption of significantly divergent cultures and social environments compared to Western countries.

The analysis will follow a two-step deductive-inductive approach. First, in the deductive step, we will extract data from papers coding the social influence sources, mechanisms (such as doctor-patient interaction, or social support), and choice constructs using the framework from Dellaert et al. (42). From this initial data extraction round, we will streamline the definitions of social influence mechanisms and choice constructs. Based on the streamlined definitions, we will inductively propose the connection of sources and mechanisms of social influence and choice constructs. Finally, we will review the initial coding scheme before completing the descriptive analysis.

From a broader perspective, the systematic literature review will support the identification of important social influences, their mechanisms, and affected choice constructs in the extant literature. This will allow the subsequent steps to exploit the signals and explore the gaps.

For peer review only

Classification: Internal

Exploratory Focus Groups and Semi-Structured Interviews

Based on the outcomes of the systematic literature review, using best-practice guidelines (43, 44), an interview guide will be developed that assesses which, and how, social influences affect healthcare user decision-making according to healthcare users. Two exploratory focus groups will be conducted to enable the further development of the content within the interview guide through discussion of issues that the research team may not have considered, to test the engagement of participants with the topic, and to assess the clarity of questions and probes in the interview guide (and make any adjustments). One focus group will be conducted with people aged 60 years and older, who were or will be offered flu vaccination and the second focus group among pregnant women who were or are choosing their birth setting. Each group will consist of 6-10 participants (43) and will be conducted in-person.

One important aspect of the focus groups is to test and further generate the interview guide. As this is a complex topic which may not be immediately understandable to participants, attention will be given to whether questions were understood by the participants (i.e., not considered too abstract in nature), whether the formulation of the questions stimulated engagement with the topic, and whether probes were clear. The focus groups may also introduce topics that were not expected, and the interview guide will be adjusted to enable these topics to be captured during individual interviews.

Subsequently, semi-structured interviews will be conducted. The semi-structured interviews will provide a detailed exploration of which, and how, social influences affect healthcare user decision-making. These interviews will be conducted among healthcare users (decision makers) and healthcare professionals in the medical fields of influenza vaccination, prostate cancer treatment, joint replacement, and birth setting. Per medical field, a minimum of 10 individuals will be recruited consisting of healthcare users (n=5) and healthcare professionals (n=5) (45). Additional interviews will be added based on whether data saturation has been reached within each medical field of interest. Interview guides will be adjusted as the analysis progresses, to take account of new and emerging findings and to pursue new lines of enquiry. Interviews will preferably be face-to-face at a convenient location for the interviewee, however if requested by the interviewee, these in-depth interviews can be conducted either via a networking facility (e.g., Microsoft Teams or Zoom) or telephone.

Participants will be recruited through already confirmed collaborations with Erasmus MC – University Medical Centre clinicians, general practices, obstetric centre (focus groups and

For peer review only - http://bmjopen.bmj.com/site/about/guidelines.xhtml

Classification: Internal

interviews), and the Prostate Centre South-West Netherlands (interviews only). Focus group participants will receive a €20 gift voucher as compensation. For the interviews, participants will receive a €10 gift voucher as compensation if they participate in a face-to-face or online interview. Focus groups and interviews will be audio-recorded, then transcribed verbatim.

Before the focus groups and in-person interviews begin, participants will be provided with information about the study, and asked to sign an informed consent form. After consent has been obtained, they will be asked to fill in a short (online) survey including questions on and their decision-making style. At the end of the interviews, participants will be presented with their survey answers and given the opportunity to revise them if they would like to do so in light of the discussion.

As part of the interviews, hierarchical mapping will be employed to construct a social proximity map (46). Participants will be asked to place influential individuals on a diagram of concentric circles, with the decision maker (i.e., healthcare user) in the middle. The placement of individuals represents the participant's social network, but also the strength of influence of each specific individual.

The analysis of the transcripts from the focus groups will be conducted prior to the interviews and will focus on the issues around the data collection process, as well as providing some preliminary themes. Transcripts from interviews will be analysed iteratively using a grounded theory constant comparative approach (47) based upon a mix of inductive and deductive (from the systematic review/initial framework) coding. The constant comparative approach compares each incident in the data with other incidents for similarities and differences and groups conceptual similarities under higher-level descriptive concepts (47). For data analysis Atlas.ti will be used. Data collected through survey and recording devices will be stored for at least ten years in the archive of Erasmus University Rotterdam library.

Delphi Method

The findings obtained in literature review and qualitative interviews will be consolidated by applying the Delphi method among experts from different medical (influenza vaccination, prostate cancer treatment, joint replacement and birth setting) and scientific (e.g., choice modelling in health economics, marketing, transportation, environmental economics) fields. Linstone & Turoff (48) introduced the Delphi method as "*a method for structuring a group communication process so that the process is effective in allowing a group of individuals, as a*

For peer review only - http://bmjopen.bmj.com/site/about/guidelines.xhtml

Classification: Internal

whole, to deal with a complex problem." The current study will utilize this method to further structure and reach consensus on the developed social interdependent choice framework.

Experts from the different fields will be proposed parts of the social interdependent choice framework and asked to provide feedback in several rounds. Experts will be contacted through email and are given a week to reply to a round of the Delphi method. After each round, a summary of the experts' perspectives will be provided to the experts, along with a revised model of social influence. This will allow the experts to revise their earlier comments.

Agreement among two-thirds (67%) of participants on a model of social influence will be considered the threshold for consensus (48). Besides consensus, group stability will also be measured. This gives an indication of consistency across rounds. A set of Chi square tests will be performed to keep track of group stability (49). If stability and consensus have both been reached, the procedure will be stopped. If after five rounds, stability or consensus has not been reached, the procedure will also be stopped. In this case, the version of the model with the most consensus will be accepted, and dissents will be noted.

Patient Involvement

Healthcare users and members of the public were not involved in the design, conduct, reporting, or dissemination of this protocol. However, experiences of healthcare users will guide the refinement of the interview guide and eventually the creation of a social interdependent choice framework in the planned study.

Discussion

This study will focus on identifying which social influences affect healthcare user decision-making, as well as the mechanisms/processes for their impacts. By forming an understanding of social influences in individual decision-making, this study will not only help to identify social influences in healthcare decision-making but also help to extend choice theory by building an evidence-based interdependent choice framework that depicts which, and how, social influences impact healthcare user decision-making. Such a framework has the potential to contribute towards more accurate predictions of healthcare choices, which could in turn have a positive impact on decreasing poor policy decisions, trial-and-error implementations, and demand-supply imbalances.

Addressing limitations

For peer review only

Classification: Internal

A limitation of the systematic literature review approach is the inclusion criteria of articles based on the study's title and abstract. This may result in missing findings regarding less straightforward mechanisms of social influences or choice constructs, that are not covered by the search strategy. Additionally, minor findings are more likely to be overlooked, as these are often not mentioned in the abstract or title. This may lead to an overrepresentation of significant results resulting from the literature review. Further validation would be necessary to estimate the effects and their significance more accurately. The current protocol is designed to (partly) correct for this: interviews and the Delphi method allow influences and influence mechanisms that are not found in the systematic literature step to surface later in the study.

Another limitation, related to the explorative focus groups and semi-structured interviews, is the recruitment of participants through healthcare providers. While on the one hand this can count as a strength due to recruiting participants who are likely experienced with healthcare decision-making within a medical field of interest, it may also lead to selection bias: these participants may not be representative of the targeted population in terms of experience with the decisions that are studied.

Ethics and Dissemination

This study was approved by the Erasmus School of Health Policy & Management Research Ethics Review Committee (ESHPM, Rotterdam, The Netherlands; reference ETH2122-0666).

All participants will be provided with an information letter and given the opportunity to ask questions on the study. Informed consent will be obtained before their participation. Data will be anonymised by removing names and any other personal information from transcripts and answers during the Delphi method.

Findings will be published in peer-reviewed academic journals and further shared with various audiences such as researchers, healthcare professionals and policy makers through methods such as public presentations and academic conferences. The gathered data and the constructed data set from the systematic literature review can be requested from the corresponding author. The full interview transcripts will not be made available for request as participants have signed a consent form that promises to maintain complete privacy.

Acknowledgement

For peer review only

Classification: Internal

The authors wish to thank Angie Fagerlin for fruitful discussion and comments on an earlier version.

Authors' Contribution

EWBG, JV, JDS, LP, JC: initial conceptualization and design of the study. All authors further adapted and improved the design of the study. SPHN: drafted the manuscript. All authors provided critical revision of the manuscript and have read and approved the final manuscript.

For peer review only

Classification: Internal

References

1. West LM, Diack L, Cordina M, Stewart D. A systematic review of the literature on “medication wastage”: an exploration of causative factors and effect of interventions. International Journal of Clinical Pharmacy. 2014 Jul 19;36(5):873–81.

2. Bloom DE, Chatterji S, Kowal P, Lloyd-Sherlock P, McKee M, Rechel B, et al. Macroeconomic implications of population ageing and selected policy responses. The Lancet [Internet]. 2015 Feb [cited 2023 Aug 4];385(9968):649–57. Available from: https://www.ncbi.nlm.nih.gov/pmc/articles/PMC4469267/

3. OECD, European Union. Health at a Glance: Europe 2018: state of health in the EU cycle. [Internet]. Brussels: OECD Publishing; 2018 [cited 2023 Jul 21]. Available from: https://doi.org/10.1787/health_glance_eur-2018-en.

4. Watkins JB. Affordability of health care: a global crisis. Value Heal. 2018;21(3):280-282.

5. Zucchelli E, Jones A, Rice N. The evaluation of health policies through microsimulation methods. Int J of Microsim, 2012;5(1):2-20.

6. Terris-Prestholt F, Quaife M, Vickerman P. Parameterising User Uptake in Economic Evaluations: The role of discrete choice experiments. Health Economics. 2016 Jan 15;25(1):116–23.

7. McFadden D. Free markets and fettered consumers. American Economic Review. 2006 Feb;96(1):5–29.

8. Hopfer S, Clippard JR. College Women’s HPV Vaccine Decision Narratives. Qualitative Health Research. 2010 Sep 14;21(2):262–77.

9. Marsh HA, Malik F, Shapiro E, Omer SB, Frew PM. Message framing strategies to increase influenza immunization uptake among pregnant African American women. Maternal and Child Health Journal. 2013 Dec 12;18(7):1639–47.

For peer review only

Classification: Internal

10. Edge R, Goodwin D, Isba R, Keegan T. Socialization, indifference, and convenience: exploring the uptake of influenza vaccine among medical students and early career doctors. Qualitative Health Research. 2017 Jul 24;27(13):1982–93.

11. Lewis S, Kenny K, Broom A, Kirby E, Boyle F. The social meanings of choice in living-with advanced breast cancer. Social Science & Medicine. 2021 Jul;280:114047.

12. de Bekker-Grob EW, Howard K, Swait J. Identifying the impact of social influences in health-related discrete choice experiments [Internet]. PLoS ONE. 2022 [cited 2023 Jul 21];17(10):e0276141. Available from: https://doi.org/10.1371/journal.pone.0276141

13. de Bekker-Grob EW, Donkers B, Bliemer M, Coast J, Swait J. Towards Accurate Prediction of Healthcare Choices: The INTERSOCIAL Project. The Patient [Internet]. 2022 Sep 1 [cited 2023 Aug 5];15(5):509–12. Available from: https://pubmed.ncbi.nlm.nih.gov/35881234/

14. Abrams D, Hogg MA. Social identification, self-categorization and social influence. European Review of Social Psychology. 1990 Jan;1(1):195–228.

15. Turner JC. Social influence. Milton Keynes: Open Univ. Press; 1991.

16. Leenders R. Modeling social influence through network autocorrelation: constructing the weight matrix. Social Networks. 2002 Jan;24(1):21–47.

17. Gass RH. Sociology of social influence. In: Wright JD, editor. International encyclopedia of the social and behavioral sciences (2nd ed). Oxford: Elsevier; 2015. p. 348–54.

18. Troiano G, Nante N, Cozzolino M. The Angelina Jolie effect – Impact on breast and ovarian cancer prevention: A systematic review of effects after the public announcement in May 2013. Health Education Journal. 2017 Jun 24;76(6):707–15.

19. Otte E, Rousseau R. Social network analysis: a powerful strategy, also for the information sciences. Journal of Information Science. 2002 Dec;28(6):441–53.

For peer review only

Classification: Internal

20. Lee SY. Cultural factors associated with breast and cervical cancer screening in Korean American women in the US: an integrative literature review. Asian Nursing Research. 2015 Jun;9(2):81–90.

21. Damiani G, Basso D, Acampora A, Bianchi CBNA, Silvestrini G, Frisicale EM, et al. The impact of level of education on adherence to breast and cervical cancer screening: Evidence from a systematic review and meta-analysis. Preventive Medicine. 2015 Dec;81:281–289.

22. Jacobs EA, Rathouz PJ, Karavolos K, Everson-Rose SA, Janssen I, Kravitz HM, et al. Perceived Discrimination Is Associated with Reduced Breast and Cervical Cancer Screening: The Study of Women's Health Across the Nation (SWAN). Journal of Women's Health. 2014 Feb;23(2):138–145.

23. Nolan JM, Schultz PW, Cialdini RB, Goldstein NJ, Griskevicius V. Normative social influence is underdetected. Personality and Social Psychology Bulletin. 2008 May 9;34(7):913–23.

24. Prabhakaran R, Gray JR. The pervasive nature of unconscious social information processing in executive control. Frontiers in Human Neuroscience. 2012;6.

25. Nederlands Jeugdinstituut. Cijfers over geboorte [Internet]. 2023 June 2 [cited 2023 Aug 4]. Available from: https://www.nji.nl/cijfers/geboorte

26. LROI. LROI 2022 [Internet]. LROI. 2022 [cited 2023 Aug 4]. Available from: https://www.lroi.nl/publicaties/jaarrapportage/

27. NKR. NKR Cijfers [Internet]. [cited 2023, Aug 8]. Available from: https://iknl.nl/nkr-cijfers

28. van Lier EA, Hament JM, Knijff M, Westra M, Ernst A, Giesbers H, et al. Vaccination coverage and annual report of the National Immunisation Programme in the Netherlands, 2022. National Institute for Public Health and the Environment. 2022 [cited 2023 Aug 4]. Available from: https://rivm.openrepository.com/handle/10029/626797

For peer review only

Classification: Internal

29. Hermus MAA, Boesveld IC, Hitzert M, Franx A, de Graaf JP, Steegers EAP, et al. Defining and describing birth centres in the Netherlands - a component study of the Dutch Birth Centre Study [Internet]. BMC Pregnancy and Childbirth. 2017 Jul 3 [cited 2023 Jul 21];17(1). Available from: https://doi.org/10.1186/s12884-017-1375-8

30. Woolf AD, Pfleger B. Burden of major musculoskeletal conditions. Bull World Health Organ. 2003;81(9):646-656.

31. Takeda R, Matsumoto T, Yuji Maenohara, Yasunori Omata, Hiroshi Inui, Nagase Y, et al. Increasing trend of radiographic features of knee osteoarthritis in rheumatoid arthritis patients before total knee arthroplasty [Internet]. Scientific Reports. 2022 Jun 21 [cited 2023 Jul 21];12(1):10452. Available from: https://doi.org/10.1038/s41598-022-14440-2

32. Bokhorst LP, Alberts AR, Rannikko A, Valdagni R, Pickles T, Kakehi Y, et al. Compliance Rates with the Prostate Cancer Research International Active Surveillance (PRIAS) Protocol and Disease Reclassification in Noncompliers. European Urology. 2015 Nov;68(5):814–21.

33. Page MJ, McKenzie JE, Bossuyt PM, Boutron I, Hoffmann TC, Mulrow CD, et al. The PRISMA 2020 statement: An updated guideline for reporting systematic reviews. British Medical Journal. 2021 Mar 29;372(71).

34. Shaller D. Patient-Centered Care: What Does It Take? Commonw Fund, 2007;68.

35. Kreling B, Figueiredo MI, Sheppard VL, Mandelblatt JS. A qualitative study of factors affecting chemotherapy use in older women with breast cancer: barriers, promoters, and implications for intervention [Internet]. Psycho-Oncology. 2006 Apr 6 [cited 2023 Jul 21];15(12):1065–76. Available from: https://doi.org/10.1002/pon.1042

36. Street RL. Information-giving in medical consultations: The influence of patients' communicative styles and personal characteristics. Social Science & Medicine. 1991 Jan;32(5):541–8.

For peer review only

Classification: Internal

37. Klassen AC, Washington C. How does social integration influence breast cancer control among urban African-American women? Results from a cross-sectional survey. BMC Women's Health. 2008 Feb 6;8(1).

38. Jones CJ, Smith H, Llewellyn C. Evaluating the effectiveness of health belief model interventions in improving adherence: A systematic review. Health Psychology Review [Internet]. 2013 Jun 12;8(3):253–69. Available from: https://www.tandfonline.com/doi/abs/10.1080/17437199.2013.802623

39. de Vries H. An Integrated Approach for Understanding Health Behavior; The I-Change Model as an Example. Psychology and Behavioral Science International Journal. 2017 Mar 9;2(2).

40. de Vries H, Mesters I, van de Steeg H, Honing C. The general public's information needs and perceptions regarding hereditary cancer: an application of the Integrated Change Model. Patient Education and Counseling. 2005 Feb;56(2):154–65.

41. Prochaska JO, Velicer WF. The Transtheoretical Model of Health Behavior Change. American Journal of Health Promotion [Internet]. 1997;12(1):38–48. Available from: https://doi.org/10.4278/0890-1171-12.1.38

42. Dellaert BGC, Swait J, Adamowicz WLV, Arentze TA, Bruch EE, Cherchi E, et al. Individuals' Decisions in the Presence of Multiple Goals [Internet]. Customer Needs and Solutions. 2017 May 11;5(1-2):51–64. Available from: https://doi.org/10.1007/s40547-017-0071-1

43. Krueger RA, Casey MA. Focus groups: a practical guide for applied research. 5th ed. Thousand Oaks, California: Sage; 2015.

44. Bryman A. Social Research Methods. 5th ed. London: Oxford University Press; 2015.

45. Creswell J. Qualitative inquiry and research design. Thousand Oaks, Calif.: Sage; 2007.

46. Antonucci T. Hierarchical mapping technique. Gen J Am Soc. 1986;10:10-2.

For peer review only

Classification: Internal

47. Strauss AL, Corbin J. Basics of qualitative research: Grounded Theory procedures and techniques. Thousand Oaks: Sage publications; 1990.

48. Linstone HA, Turoff M. The Delphi Method: Techniques and Applications. Technometrics. 1975 Aug;18(3):363.

49. von der Gracht HA. Consensus measurement in Delphi studies. Technological Forecasting and Social Change. 2012 Oct;79(8):1525–36.

For peer review only

Classification: Internal

Standards for Reporting Qualitative Research (SRQR)*

http://www.equator-network.org/reporting-guidelines/srqr/

Page/line no(s).

Title and abstract

**Title** - Concise description of the nature and topic of the study Identifying the study as qualitative or indicating the approach (e.g., ethnography, grounded theory) or data collection methods (e.g., interview, focus group) is recommended	Title page
**Abstract** - Summary of key elements of the study using the abstract format of the intended publication; typically includes background, purpose, methods, results, and conclusions	Abstract (p. 3)

Introduction

**Problem formulation** - Description and significance of the problem/phenomenon studied; review of relevant theory and empirical work; problem statement	Introduction (p. 5)
**Purpose or research question** - Purpose of the study and specific objectives or questions	Objectives (p. 5-6)

Methods

**Qualitative approach and research paradigm** - Qualitative approach (e.g., ethnography, grounded theory, case study, phenomenology, narrative research) and guiding theory if appropriate; identifying the research paradigm (e.g., postpositivist, constructivist/ interpretivist) is also recommended; rationale**	Described on p. 8-12 per section of planned study.
**Researcher characteristics and reflexivity** - Researchers' characteristics that may influence the research, including personal attributes, qualifications/experience, relationship with participants, assumptions, and/or presuppositions; potential or actual interaction between researchers' characteristics and the research questions, approach, methods, results, and/or transferability	Not included in the protocol, but will be included in publications resulting from the planned study.
**Context** - Setting/site and salient contextual factors; rationale**	p. 10 (interviews) and p. 12 (Delphi method)
**Sampling strategy** - How and why research participants, documents, or events were selected; criteria for deciding when no further sampling was necessary (e.g., sampling saturation); rationale**	Planned recruitment strategies for interviews are described on p. 10-11
**Ethical issues pertaining to human subjects** - Documentation of approval by an appropriate ethics review board and participant consent, or explanation for lack thereof; other confidentiality and data security issues	Ethics and Dissemination (p. 13)

For peer review only - http://bmjopen.bmj.com/site/about/guidelines.xhtml

Classification: Internal

**Data collection methods** - Types of data collected; details of data collection procedures including (as appropriate) start and stop dates of data collection and analysis, iterative process, triangulation of sources/methods, and modification of procedures in response to evolving study findings; rationale**	Described in Research Design for each of the three steps (p. 6-12).
**Data collection instruments and technologies** - Description of instruments (e.g., interview guides, questionnaires) and devices (e.g., audio recorders) used for data collection; if/how the instrument(s) changed over the course of the study	Described in Research Design for each of the three steps (p. 6-12).
**Units of study** - Number and relevant characteristics of participants, documents, or events included in the study; level of participation (could be reported in results)	Described in Research Design for each of the three steps (p. 6-12).
**Data processing** - Methods for processing data prior to and during analysis, including transcription, data entry, data management and security, verification of data integrity, data coding, and anonymization/de-identification of excerpts	Described in Research Design for each of the three steps (p. 6-12).
**Data analysis** - Process by which inferences, themes, etc., were identified and developed, including the researchers involved in data analysis; usually references a specific paradigm or approach; rationale**	Described in Research Design for each of the three steps (p. 6-12).
**Techniques to enhance trustworthiness** - Techniques to enhance trustworthiness and credibility of data analysis (e.g., member checking, audit trail, triangulation); rationale**	Described in Research Design for each of the three steps (iterative mixed-method approach; p. 6-12).

Results/findings

**Synthesis and interpretation** - Main findings (e.g., interpretations, inferences, and themes); might include development of a theory or model, or integration with prior research or theory	N/A (outcomes of proposed study are described in Objectives section (p. 5-6))
**Links to empirical data** - Evidence (e.g., quotes, field notes, text excerpts, photographs) to substantiate analytic findings	N/A

Discussion

For peer review only - http://bmjopen.bmj.com/site/about/guidelines.xhtml

Classification: Internal

**Integration with prior work, implications, transferability, and contribution(s) to the field -** Short summary of main findings; explanation of how findings and conclusions connect to, support, elaborate on, or challenge conclusions of earlier scholarship; discussion of scope of application/generalizability; identification of unique contribution(s) to scholarship in a discipline or field	N/A
**Limitations** - Trustworthiness and limitations of findings	N/A

Other

**Conflicts of interest** - Potential sources of influence or perceived influence on study conduct and conclusions; how these were managed	Title page: Competing Statement
**Funding** - Sources of funding and other support; role of funders in data collection, interpretation, and reporting	Title page: Funding Statement

*The authors created the SRQR by searching the literature to identify guidelines, reporting standards, and critical appraisal criteria for qualitative research; reviewing the reference lists of retrieved sources; and contacting experts to gain feedback. The SRQR aims to improve the transparency of all aspects of qualitative research by providing clear standards for reporting qualitative research.

**The rationale should briefly discuss the justification for choosing that theory, approach, method, or technique rather than other options available, the assumptions and limitations implicit in those choices, and how those choices influence study conclusions and transferability. As appropriate, the rationale for several items might be discussed together.

Reference:
O'Brien BC, Harris IB, Beckman TJ, Reed DA, Cook DA. **Standards for reporting qualitative research: a synthesis of recommendations.** *Academic Medicine*, Vol. 89, No. 9 / Sept 2014 DOI: 10.1097/ACM.0000000000000388

For peer review only

Classification: Internal

BMJ Open

A Socially Interdependent Choice Framework for Social Influences in Healthcare Decision-Making: A Study Protocol

Journal:	*BMJ Open*
Manuscript ID	bmjopen-2023-079768.R1
Article Type:	Protocol
Date Submitted by the Author:	20-Dec-2023

Complete List of Authors:	Nouwens, Sven; Erasmus Universiteit Rotterdam Erasmus School of Health Policy and Management; Erasmus University Rotterdam, Erasmus Choice Modeling Centre<br>Veldwijk, Jorien; Erasmus Universiteit Rotterdam Erasmus School of Health Policy and Management; Erasmus University Rotterdam, Erasmus Choice Modeling Centre<br>Pilli, Luis; Erasmus Universiteit Rotterdam Erasmus School of Health Policy and Management; Erasmus University Rotterdam, Erasmus Choice Modeling Centre<br>Swait, J.D.; Erasmus Universiteit Rotterdam Erasmus School of Health Policy and Management; Erasmus University Rotterdam, Erasmus Choice Modeling Centre<br>Coast, Joanna; University of Bristol, Population Health Sciences<br>de Bekker-Grob, Esther; Erasmus Universiteit Rotterdam Erasmus School of Health Policy and Management; Erasmus University Rotterdam, Erasmus Choice Modeling Centre

**Primary Subject Heading**:	Health economics
Secondary Subject Heading:	Health economics, Health policy
Keywords:	Social Interaction, HEALTH ECONOMICS, Social Support

SCHOLARONE™
Manuscripts

A Socially Interdependent Choice Framework for Social Influences in Healthcare Decision-Making: A Study Protocol

S.P.H. Nouwens[1,2,3*], J. Veldwijk[1,2,3], L. Pilli[1,2,3], J.D. Swait[1,2], J. Coast[4], E.W. de Bekker-Grob[1,2,3]

[1] Erasmus School of Health Policy & Management, Erasmus University Rotterdam

[2] Erasmus Choice Modeling Centre, Erasmus University Rotterdam, the Netherlands

3 Erasmus Centre for Health Economics Rotterdam, Erasmus University, the Netherlands

[4] Health Economics Bristol, Population Health Sciences, Bristol Medical School, University of Bristol, UK

* Correspondence to:

S.P.H. Nouwens

Erasmus School of Health Policy & Management

Erasmus University Rotterdam

P.O. Box 1738

3000 DR Rotterdam

The Netherlands

nouwens@eshpm.eur.nl

Funding Statement

The project has received funding from the Dutch Research Council (NWO-Talent-Scheme-Vidi-Grant No. 09150171910002). Joanna Coast is supported by the Wellcome Trust [205384/Z/16/Z]. For the purpose of open access, the author has applied a CC BY public copyright license to any Author Accepted Manuscript version arising from this submission.

Competing Statement

All authors have completed the ICMJE uniform disclosure form at http://www.icmje.org/disclosure-of-interest/ and declare: no support from any organisation for the submitted work; no financial relationships with any organisations that might have an

For peer review only

Classification: Internal

interest in the submitted work in the previous three years; no other relationships or activities that could appear to have influenced the submitted work.

Word Count: 3532

Keywords: Social Interaction, Social Environment, Decision-Making, Patient Preferences, Choice Behaviour

For peer review only

Classification: Internal

Abstract

Objectives Current choice models in healthcare (and beyond) can provide suboptimal predictions of healthcare users' decisions. One reason for such inaccuracy is that standard microeconomic theory assumes that decisions of healthcare users are made in a social vacuum. Healthcare choices, however, can in fact be (entirely) socially determined. To achieve more accurate choice predictions within healthcare and therefore better policy-decisions, the social influences that affect healthcare user decision-making need to be identified and explicitly integrated into choice models. The purpose of this study is to develop a socially interdependent choice framework of healthcare user decision-making.

Design A mixed methods approach will be used. A systematic literature review will be conducted that identifies the social influences on healthcare user decision-making. Based on the outcomes of a systematic literature review, an interview guide will be developed that assesses which, and how, social influences affect healthcare user decision-making in four different medical fields. This guide will be used during two exploratory focus groups to assess the engagement of participants and clarity of questions and probes. The refined interview guide will be used to conduct the semi-structured interviews with healthcare professionals and users. These interviews will explore in detail which, and how, social influences affect healthcare user decision-making. Focus group and interview transcripts will be analysed iteratively using a constant comparative approach based upon a mix of inductive and deductive coding. Based on the outcomes a social influence independent choice framework for healthcare user decision-making will be drafted. Finally, the Delphi technique will be employed to achieve consensus about the final version of this choice framework.

Ethics and dissemination This study was approved by the Erasmus School of Health Policy & Management Research Ethics Review Committee (ESHPM, Rotterdam, The Netherlands; reference ETH2122-0666).

Strengths and weaknesses of this study

- This is the first study that will attempt to develop a socially interdependent choice framework of healthcare user decision-making.
- Through applying a mixed method approach, existing knowledge and empirical evidence will be combined to ensure a rigorous approach for framework development.

For peer review only

Classification: Internal

- Inclusion of articles based on the study's title and abstract in the systematic literature review step may result in potentially relevant areas remaining unexamined. In part, this will be corrected in the interviews and Delphi method steps, which allow influences and influence mechanisms to surface later in the study.
- Participant recruitment via healthcare providers may lead to selection bias. However, recruited participants will likely be experienced with healthcare decision-making within a medical field of interest.

For peer review only

Classification: Internal

Introduction

Rising expenditures, an ageing population, high prices of new medical treatment options, and substantial annual resource waste put healthcare under heavy pressure (1-4). To avoid poor policy decisions, trial-and-error implementation and demand-supply imbalance, there is a need for accurate predictions of healthcare users' choice behaviour (5, 6). Current choice models in healthcare (and beyond), however, provide inaccurate predictions of individual healthcare user decisions. One reason for such inaccuracy is that standard microeconomic theory assumes that decision-making occurs in a social vacuum. Choice behaviour, under this *homo economicus* perspective (7), is regarded as a completely rational process in which the decision maker is driven purely by their own interest, disregarding any self- or other-originating psychological factors such as social influences. However, healthcare user choices can in fact be (entirely) socially determined (8-12). To achieve more accurate choice behaviour predictions within healthcare and therefore better policy-decisions, the social influences that affect healthcare users' decision-making need to be identified and explicitly integrated into choice models.

This motivates the need for an expanded choice paradigm that must be developed and validated. In the pursuit of this objective, a first important step is to develop and extend the theory of decision-making by building a conceptual framework that depicts which social influences impact healthcare user decision-making, and how they achieve their impact. With this in mind, we developed a systematic approach that 1) empirically identifies these social influences and 2) suggests mechanisms for their impacts. We strive towards these objectives using a phased approach: a systematic literature review, a series of focus groups and semi-structured interviews, and the Delphi method will supplement each other. This paper provides the protocol for the systematic approach that will be taken towards developing this socially interdependent choice framework.

Objectives

This study is part of the INTERSOCIAL project that aims to develop and validate a socially interdependent choice paradigm for healthcare user decision-making such that more accurate predictions of choice behaviour in healthcare can be achieved (13). This study protocol will focus on conducting the necessary steps that will guide the attempt to extend the existing theoretical choice framework to account for social influences. The objectives for this study protocol are:

For peer review only - http://bmjopen.bmj.com/site/about/guidelines.xhtml
Classification: Internal

1. to identify which, and how, social influences have previously been found to affect healthcare user decision-making based on existing published peer-reviewed studies;
2. to explore with healthcare users in four different areas which, and how, social influences affect healthcare user decision-making; and
3. to develop consensus among experts (including healthcare professionals) about which, and how, social influences affect healthcare user decision-making.

Definition of Social Influence

There exist many definitions of social influence (14-17) with different levels of practical applicability. Based on a consensus meeting with the research team and external experts (n=16) from different academic fields, it was decided to use the following definition in the present study: "*social influence* is the process by which an individual's behaviour, thoughts, beliefs, attitudes, feelings, actions, preferences, decisions, or choice behaviour are (directly and/or indirectly) affected by other people." These 'other people' can be anyone that the decision maker (in our case, users of healthcare) is in direct contact with but also individuals that the decision maker is not in direct contact with, e.g., celebrity endorsements (18).

Note that our definition is centred on a decision maker whose healthcare decisions may depend upon other people in the decision maker's social circle, implying that we are excluding the study of networks of individual healthcare users who are jointly influenced by each other (19). We apply a unidirectional paradigm where the entire social network is centred on a single healthcare user and study how this social network ((types of) individuals and processes) influences the healthcare user, but not how the healthcare user influences others' social networks or how a healthcare user's social contacts are influenced by each other. We only include social influences that can be tracked to a single source. Other social influences (such as culture (20), education (21) and perceived discrimination (22)) that cannot be tracked to a single source will be documented only.

Research Design

The research process will use a mixed-methods approach. First, a systematic literature review that identifies the (types of) individuals and the processes that systematically influence healthcare users' decisions within different medical fields will be performed. The results from the systematic literature review will aid in constructing an interview guide for the subsequent round of (group and individual) interviews. As individuals are often unconscious and unaware

For peer review only

Classification: Internal

of the degree to which they are socially influenced (23-24), the results from the systematic literature review can additionally help to construct interview questions that stimulate recall and awareness of social influences that might otherwise not be raised by respondents. Second, two focus groups and forty semi-structured interviews will be conducted to explore and assess healthcare users' and healthcare professionals' views on social influences on healthcare user decision-making. The focus groups have a secondary aim of testing engagement of participants with the topic and assessing the clarity of questions and probes in the interview guide. Their feedback will then be used to refine the interview guide for the semi-structured interviews. Third, the Delphi method will be applied to reach consensus among experts (including healthcare professionals and choice modellers) about the socially interdependent choice framework on healthcare user decision-making.

For the systematic literature review the following medical fields of study have been chosen: vaccination, birth setting, arthroplasty, and prostate cancer treatment. The rationale for choosing these fields is fourfold. First, healthcare users in these fields face a degree of autonomy in their choice, meaning that they are not obliged to choose one alternative over the other, which is essential for testing real choice behaviour predictability. Second, many healthcare users face these medical fields (in the Netherlands, which has 17.5 million inhabitants: almost all people are affected by vaccinations, between 160,000 and 180,000 people give birth yearly (25), 31,514 hip replacements were reported in 2022 (26), and prevalence of prostate cancer is over 55,000 (27)) speaking to relevance from the perspective of development of an extended socially interdependent framework. Third, a higher degree of generalizability is achieved by including medical fields that are likely to differ in types/degrees of social interdependence and consequences that may employ different mechanisms of social influence. Fourth, in each of these four fields, there are current policy challenges related to the decisions made by healthcare users in these areas: i) the demand for influenza vaccination is lower than for other vaccinations (28), ii) there is increasing interest in birth centres in the Dutch population (29), iii) there is an exponential rise in patients with joint problems (30-31), and iv) there is an alarming decrease in active-surveillance compliance from prostate cancer patients (32-33).

Systematic Literature Review

Initially, a systematic literature review of social influences on healthcare user decision-making will be performed. The review will follow PRISMA guidelines (34) and map the

For peer review only - http://bmjopen.bmj.com/site/about/guidelines.xhtml

Classification: Internal

literature in three dimensions that concern the development of the socially interdependent choice paradigm proposed by the INTERSOCIAL project (13). These dimensions describe i) the individuals or groups influencing health-related decision-making; ii) the mechanisms through which they influence decisions of healthcare users; and iii) the choice constructs that these processes affect. A narrative synthesis of the sources and mechanisms of social influence and choice constructs affected will be provided.

Concerning the first dimension, psychosocial variables are one of the key facets of patient-centred care (35). The past literature offers evidence of treatment choices as an action embedded in a network of social relationships, including experts and non-experts (36-37). This first dimension focuses on which individuals' and groups' actions must be considered for the development of the theoretical framework for healthcare user decision-making.

The second dimension, i.e., social influence mechanisms, refers to the processes by which a healthcare user's behaviour, thoughts, beliefs, attitudes, feelings, actions, preferences, decisions, or choice behaviours are (directly and/or indirectly) affected by other people (38-39). Mechanisms and dimensions already present in the health-related literature will support classifying included papers into categories. Physician-patient interaction (36-37) and social support (40) are two relevant and well-defined constructs that may impact healthcare user decision-making. Social norm is a relevant construct from socio-cognitive theories, and it is present in many attitudinal-behavioural models in the health domain, such as the Health Belief (41) model, I-Change (42-43) model, and the Transtheoretical Model of Behavior Change (44).

The creation of a socially interdependent choice framework demands the connection between the first two dimensions and the choice constructs involved in choice econometric models, such as the choice framework of Dellaert et al. (45), which describes how individuals evaluate attributes and make decisions in the presence of multiple goals. Since insights about this connection from the extant literature are sparse at best, choice constructs will be aggregated into three broadly defined main categories. These categories contain the effects of social influences on (1) healthcare users' goals, (2) preference formation, and (3) non-evaluative components of choice models. Goals involve crucial personal trade-offs such as life expectancy versus quality of life, where different alternatives are valued differently based on the goal that a healthcare user seeks to attain, with other persons possibly influencing the activation of certain goals. Preference formation describes the effects of social influences on

For peer review only

Classification: Internal

the acquisition of information about attributes and the deployment of weights to these attributes to determine options' total utilities. Finally, non-evaluative components refer to any steps of the decision-making process where certain options are excluded based on single attributes, instead of an option's unique blend of attribute levels. Examples of non-evaluative components are habitual behaviour, non-compensatory processes, or screening of alternatives before evaluation.

To attain the objectives of the systematic literature review, nine different databases will be searched: Embase, Cochrane CENTRAL, Econlit, Google Scholar, International Bibliography of the Social Sciences, Medline, Psychinfo, Scopus, and Web of Science. To be eligible, a paper must return from a search containing (variations around) the keywords "social influence" AND "decision-making" AND "empirical data" AND "healthcare field". The "healthcare field" differs for each of the four healthcare fields studied. For example, the field of vaccination will use the keywords "vaccine" OR "vaccine hesitancy" OR "vaccination" OR "vaccination coverage". We will exclude the terms "parental" and "parental decision-making", as we are interested in how a healthcare user makes choices about their own health and how social influences affect these choices.

The inclusion criteria further require that the full text of a paper is accessible, is written in English, and presents results of original empirical data collected in Western countries. Countries that will be included in the search strategy are the United States, Canada, North America, United Kingdom, England, Ireland, Northern Ireland, Scotland, The Netherlands, Belgium, France, Germany, Denmark, Norway, Sweden, Finland, Spain, Portugal, Italy, Austria, Switzerland, Iceland, Luxembourg, Liechtenstein, San Marino, Monaco, Andorra, Malta, Cyprus, Gibraltar, Europe, Australia, and New Zealand. Papers reporting data from other countries will be excluded due to the assumption of significantly divergent cultures and social environments compared to Western countries.

The analysis will follow a two-step deductive-inductive approach. First, in the deductive step, we will extract data from papers coding the social influence sources, mechanisms (such as doctor-patient interaction, or social support), and choice constructs using the framework from Dellaert et al. (45). From this initial data extraction round, we will streamline the definitions of social influence mechanisms and choice constructs. Based on the streamlined definitions, we will inductively propose the connection of sources and mechanisms of social influence and choice constructs. The last step of the process is to describe frequencies and co-occurrences of

For peer review only - http://bmjopen.bmj.com/site/about/guidelines.xhtml

Classification: Internal

relevant individuals and groups, social influence mechanisms and choice constructs, and differences across medical areas.

From a broader perspective, the systematic literature review will support the identification of important social influences, their mechanisms, and affected choice constructs in the extant literature. This will allow the subsequent steps to exploit the signals and explore the gaps.

Exploratory Focus Groups and Semi-Structured Interviews

Based on the outcomes of the systematic literature review, using best-practice guidelines (46, 47), an interview guide will be developed that assesses which, and how, social influences affect healthcare user decision-making according to healthcare users. Two exploratory focus groups will be conducted to enable the further development of the content within the interview guide through discussion of issues that the research team may not have considered, to test the engagement of participants with the topic, and to assess the clarity of questions and probes in the interview guide (and make any adjustments). One focus group will be conducted with people aged 60 years and older, who were or will be offered flu vaccination and the second focus group among pregnant women who were or are choosing their birth setting. Each group will consist of 6-10 participants (46) and will be conducted in-person.

One important aspect of the focus groups is to test and further generate the interview guide. As this is a complex topic which may not be immediately understandable to participants, attention will be given to whether questions were understood by the participants (i.e., not considered too abstract in nature), whether the formulation of the questions stimulated understanding of the topic, and whether probes were clear. The focus groups may also introduce topics that were not expected, and the interview guide will be adjusted to enable these topics to be captured during individual interviews.

Subsequently, semi-structured interviews will be conducted. The semi-structured interviews will provide a detailed exploration of which, and how, social influences affect healthcare user decision-making. These interviews will be conducted among healthcare users (decision makers) and healthcare professionals in the medical fields of influenza vaccination, prostate cancer treatment, joint replacement, and birth setting. Per medical field, a minimum of 10 individuals will be recruited consisting of healthcare users (n=5) and healthcare professionals (n=5) (48). Additional interviews will be added based on whether data saturation has been reached within each medical field of interest. Interview guides will be adjusted as the analysis

For peer review only

Classification: Internal

progresses, to take account of new and emerging findings and to pursue new lines of enquiry. Interviews will preferably be face-to-face at a convenient location for the interviewee, however if requested by the interviewee, these in-depth interviews can be conducted either via a networking facility (e.g., Microsoft Teams or Zoom) or telephone.

Participants will be recruited through already confirmed collaborations with Erasmus MC – University Medical Centre clinicians, general practices, obstetric centre (focus groups and interviews), and the Prostate Centre South-West Netherlands (interviews only). Focus group participants will receive a €20 gift voucher as compensation. For the interviews, participants will receive a €10 gift voucher as compensation if they participate in a face-to-face or online interview. Focus groups and interviews will be audio-recorded, then transcribed verbatim.

Before the focus groups and in-person interviews begin, participants will be provided with information about the study, and asked to sign an informed consent form. After consent has been obtained, they will be asked to fill in a short (online) survey including questions on their health literacy (49) and their decision-making style (50). At the end of the interviews, participants will be presented with their survey answers and given the opportunity to revise them if they would like to do so in light of the discussion.

As part of the interviews, hierarchical mapping will be employed to construct a social proximity map (51). Participants will be asked to place influential individuals on a diagram of concentric circles, with the decision maker (i.e., healthcare user) in the middle. The placement of individuals represents the participant's social network, but also the strength of influence of each specific individual.

The analysis of the transcripts from the focus groups will be conducted prior to the interviews and will focus on the issues around the data collection process, as well as providing some preliminary themes. Transcripts from interviews will be analysed iteratively using a grounded theory constant comparative approach (52) based upon a mix of inductive and deductive (from the systematic review/initial framework) coding. The constant comparative approach compares each incident in the data with other incidents for similarities and differences and groups conceptual similarities under higher-level descriptive concepts (52). The analysis will result in a first version of the theoretical framework of social influence on healthcare user decision-making. For data analysis Atlas.ti will be used. Data collected through survey and recording devices will be stored for at least ten years through SURF Yoda, in line with Erasmus University policy.

For peer review only - http://bmjopen.bmj.com/site/about/guidelines.xhtml

Classification: Internal

Delphi Method

The findings obtained in the literature review and qualitative interviews will be consolidated by applying the Delphi method among experts from different medical (influenza vaccination, prostate cancer treatment, joint replacement and birth setting) and scientific (e.g., choice modelling in health economics, marketing, transportation, environmental economics) fields. Linstone & Turoff (53) introduced the Delphi method as "*a method for structuring a group communication process so that the process is effective in allowing a group of individuals, as a whole, to deal with a complex problem.*" The current study will utilize this method to further structure and reach consensus on the developed socially interdependent choice framework.

Experts (researchers and clinicians) from the different fields will be proposed parts of the socially interdependent choice framework and asked to provide feedback in several rounds. Experts will be contacted through email and are given a week to reply to a round of the Delphi method. After each round, a summary of the experts' perspectives will be provided to the experts, along with a revised model of social influence. This will allow the experts to revise their earlier comments.

Agreement among two-thirds (67%) of participants on a model of social influence will be considered the threshold for consensus (53). Besides consensus, group stability will also be measured. This gives an indication of consistency across rounds. A set of Chi square tests will be performed to keep track of group stability (54). If stability and consensus have both been reached, the procedure will be stopped. If after five rounds, stability or consensus has not been reached, the procedure will also be stopped. In this case, the version of the socially interdependent framework with the most consensus will be accepted, and dissents will be noted.

Patient Involvement

Healthcare users and members of the public were not involved in the design, conduct, reporting, or dissemination of this protocol. However, experiences of healthcare users will guide the refinement of the interview guide and eventually the creation of a socially interdependent choice framework in the planned study.

Discussion

For peer review only

Classification: Internal

This study will focus on identifying which social influences affect healthcare user decision-making, as well as the mechanisms/processes for their impacts. By forming an understanding of social influences in individual decision-making, this study will not only help to identify social influences in healthcare decision-making but also help to extend choice theory by building an evidence-based interdependent choice framework that depicts which, and how, social influences impact healthcare user decision-making. By incorporating these social influences, which can have a big impact on the choice process (8-12), such a framework has the potential to contribute towards more accurate predictions of healthcare choices, which could in turn have a positive impact on decreasing poor policy decisions, trial-and-error implementations, and demand-supply imbalances.

Addressing limitations

A limitation of the systematic literature review approach is the inclusion criteria of articles based on the study's title and abstract. This may result in missing findings regarding less straightforward mechanisms of social influences or choice constructs, that are not covered by the search strategy. Additionally, minor findings are more likely to be overlooked, as these are often not mentioned in the abstract or title. This may lead to an overrepresentation of significant results resulting from the literature review. Further validation would be necessary to estimate the effects and their significance more accurately. The current protocol is designed to (partly) correct for this: interviews and the Delphi method allow influences and influence mechanisms that are not found in the systematic literature step to surface later in the study.

Another limitation, related to the explorative focus groups and semi-structured interviews, is the recruitment of participants through healthcare providers. While on the one hand this can count as a strength due to recruiting participants who are likely experienced with healthcare decision-making within a medical field of interest, it may also lead to selection bias: these participants may not be representative of the targeted population in terms of experience with the decisions that are studied.

Ethics and Dissemination

This study was approved by the Erasmus School of Health Policy & Management Research Ethics Review Committee (ESHPM, Rotterdam, The Netherlands; reference ETH2122-0666).

All participants will be provided with an information letter and given the opportunity to ask questions on the study. Informed consent will be obtained before their participation. Data will

For peer review only

Classification: Internal

be anonymised by removing names and any other personal information from transcripts and answers during the Delphi method.

Findings will be published in peer-reviewed academic journals and further shared with various audiences such as researchers, healthcare professionals and policy makers through methods such as public presentations and academic conferences. The gathered data and the constructed data set from the systematic literature review can be requested from the corresponding author. The full interview transcripts will not be made available for request as participants have signed a consent form that promises to maintain complete privacy.

Acknowledgement

The authors wish to thank Angie Fagerlin for fruitful discussion and comments on an earlier version.

Authors' Contribution

EWBG, JV, JDS, LP, JC: initial conceptualization and design of the study. All authors further adapted and improved the design of the study. SPHN: drafted the manuscript. All authors provided critical revision of the manuscript and have read and approved the final manuscript.

For peer review only

Classification: Internal

References

1. West LM, Diack L, Cordina M, Stewart D. A systematic review of the literature on "medication wastage": an exploration of causative factors and effect of interventions. International Journal of Clinical Pharmacy. 2014 Jul 19;36(5):873–81.

2. Bloom DE, Chatterji S, Kowal P, Lloyd-Sherlock P, McKee M, Rechel B, et al. Macroeconomic implications of population ageing and selected policy responses. The Lancet [Internet]. 2015 Feb [cited 2023 Aug 4];385(9968):649–57. Available from: https://www.ncbi.nlm.nih.gov/pmc/articles/PMC4469267/

3. OECD, European Union. Health at a Glance: Europe 2018: state of health in the EU cycle. [Internet]. Brussels: OECD Publishing; 2018 [cited 2023 Jul 21]. Available from: https://doi.org/10.1787/health_glance_eur-2018-en.

4. Watkins JB. Affordability of health care: a global crisis. Value Heal. 2018;21(3):280-282.

5. Zucchelli E, Jones A, Rice N. The evaluation of health policies through microsimulation methods. Int J of Microsim, 2012;5(1):2-20.

6. Terris-Prestholt F, Quaife M, Vickerman P. Parameterising User Uptake in Economic Evaluations: The role of discrete choice experiments. Health Economics. 2016 Jan 15;25(1):116–23.

7. McFadden D. Free markets and fettered consumers. American Economic Review. 2006 Feb;96(1):5–29.

8. Hopfer S, Clippard JR. College Women's HPV Vaccine Decision Narratives. Qualitative Health Research. 2010 Sep 14;21(2):262–77.

9. Marsh HA, Malik F, Shapiro E, Omer SB, Frew PM. Message framing strategies to increase influenza immunization uptake among pregnant African American women. Maternal and Child Health Journal. 2013 Dec 12;18(7):1639–47.

For peer review only

Classification: Internal

10. Edge R, Goodwin D, Isba R, Keegan T. Socialization, indifference, and convenience: exploring the uptake of influenza vaccine among medical students and early career doctors. Qualitative Health Research. 2017 Jul 24;27(13):1982–93.

11. Lewis S, Kenny K, Broom A, Kirby E, Boyle F. The social meanings of choice in living-with advanced breast cancer. Social Science & Medicine. 2021 Jul;280:114047.

12. de Bekker-Grob EW, Howard K, Swait J. Identifying the impact of social influences in health-related discrete choice experiments [Internet]. PLoS ONE. 2022 [cited 2023 Jul 21];17(10):e0276141. Available from: https://doi.org/10.1371/journal.pone.0276141

13. de Bekker-Grob EW, Donkers B, Bliemer M, Coast J, Swait J. Towards Accurate Prediction of Healthcare Choices: The INTERSOCIAL Project. The Patient [Internet]. 2022 Sep 1 [cited 2023 Aug 5];15(5):509–12. Available from: https://pubmed.ncbi.nlm.nih.gov/35881234/

14, Abrams D, Hogg MA. Social identification, self-categorization and social influence. European Review of Social Psychology. 1990 Jan;1(1):195–228.

15. Turner JC. Social influence. Milton Keynes: Open Univ. Press; 1991.

16. Leenders R. Modeling social influence through network autocorrelation: constructing the weight matrix. Social Networks. 2002 Jan;24(1):21–47.

17. Gass RH. Sociology of social influence. In: Wright JD, editor. International encyclopedia of the social and behavioral sciences (2nd ed). Oxford: Elsevier; 2015. p. 348–54.

18. Troiano G, Nante N, Cozzolino M. The Angelina Jolie effect – Impact on breast and ovarian cancer prevention: A systematic review of effects after the public announcement in May 2013. Health Education Journal. 2017 Jun 24;76(6):707–15.

19. Otte E, Rousseau R. Social network analysis: a powerful strategy, also for the information sciences. Journal of Information Science. 2002 Dec;28(6):441–53.

For peer review only - http://bmjopen.bmj.com/site/about/guidelines.xhtml

Classification: Internal

20. Lee SY. Cultural factors associated with breast and cervical cancer screening in Korean American women in the US: an integrative literature review. Asian Nursing Research. 2015 Jun;9(2):81–90.

21. Damiani G, Basso D, Acampora A, Bianchi CBNA, Silvestrini G, Frisicale EM, et al. The impact of level of education on adherence to breast and cervical cancer screening: Evidence from a systematic review and meta-analysis. Preventive Medicine. 2015 Dec;81:281–289.

22. Jacobs EA, Rathouz PJ, Karavolos K, Everson-Rose SA, Janssen I, Kravitz HM, et al. Perceived Discrimination Is Associated with Reduced Breast and Cervical Cancer Screening: The Study of Women's Health Across the Nation (SWAN). Journal of Women's Health. 2014 Feb;23(2):138–145.

23. Nolan JM, Schultz PW, Cialdini RB, Goldstein NJ, Griskevicius V. Normative social influence is underdetected. Personality and Social Psychology Bulletin. 2008 May 9;34(7):913–23.

24. Prabhakaran R, Gray JR. The pervasive nature of unconscious social information processing in executive control. Frontiers in Human Neuroscience. 2012;6.

25. Nederlands Jeugdinstituut. Cijfers over geboorte [Internet]. 2023 June 2 [cited 2023 Aug 4]. Available from: https://www.nji.nl/cijfers/geboorte

26. LROI. LROI 2022 [Internet]. LROI. 2022 [cited 2023 Aug 4]. Available from: https://www.lroi.nl/publicaties/jaarrapportage/

27. NKR. NKR Cijfers [Internet]. [cited 2023, Aug 8]. Available from: https://iknl.nl/nkr-cijfers

28. van Lier EA, Hament JM, Knijff M, Westra M, Ernst A, Giesbers H, et al. Vaccination coverage and annual report of the National Immunisation Programme in the Netherlands, 2022. National Institute for Public Health and the Environment. 2022 [cited 2023 Aug 4]. Available from: https://rivm.openrepository.com/handle/10029/626797

For peer review only

Classification: Internal

29. Hermus MAA, Boesveld IC, Hitzert M, Franx A, de Graaf JP, Steegers EAP, et al. Defining and describing birth centres in the Netherlands - a component study of the Dutch Birth Centre Study [Internet]. BMC Pregnancy and Childbirth. 2017 Jul 3 [cited 2023 Jul 21];17(1). Available from: https://doi.org/10.1186/s12884-017-1375-8

30. Woolf AD, Pfleger B. Burden of major musculoskeletal conditions. Bull World Health Organ. 2003;81(9):646-656.

31. Takeda R, Matsumoto T, Yuji Maenohara, Yasunori Omata, Hiroshi Inui, Nagase Y, et al. Increasing trend of radiographic features of knee osteoarthritis in rheumatoid arthritis patients before total knee arthroplasty [Internet]. Scientific Reports. 2022 Jun 21 [cited 2023 Jul 21];12(1):10452. Available from: https://doi.org/10.1038/s41598-022-14440-2

32. Bokhorst LP, Alberts AR, Rannikko A, Valdagni R, Pickles T, Kakehi Y, et al. Compliance Rates with the Prostate Cancer Research International Active Surveillance (PRIAS) Protocol and Disease Reclassification in Noncompliers. European Urology. 2015 Nov;68(5):814–21.

33. Kinsella N, Stattin P, Cahill D, Brown C, Bill-Axelson A, Bratt O, et al. Factors Influencing Men's Choice of and Adherence to Active Surveillance for Low-risk Prostate Cancer: A Mixed-method Systematic Review. Eur Urol. 2018;74(3):261-280. doi:10.1016/j.eururo.2018.02.026

34. Page MJ, McKenzie JE, Bossuyt PM, Boutron I, Hoffmann TC, Mulrow CD, et al. The PRISMA 2020 statement: An updated guideline for reporting systematic reviews. British Medical Journal. 2021 Mar 29;372(71).

35. Shaller D. Patient-Centered Care: What Does It Take? Commonw Fund, 2007;68.

36. Kreling B, Figueiredo MI, Sheppard VL, Mandelblatt JS. A qualitative study of factors affecting chemotherapy use in older women with breast cancer: barriers, promoters, and implications for intervention [Internet]. Psycho-Oncology. 2006 Apr 6 [cited 2023 Jul 21];15(12):1065–76. Available from: https://doi.org/10.1002/pon.1042

For peer review only

Classification: Internal

37. Street RL. Information-giving in medical consultations: The influence of patients' communicative styles and personal characteristics. Social Science & Medicine. 1991 Jan;32(5):541–8.

38. House JS, Landis KR, Umberson D. Social relationships and health. Science. 1988;241(4865):540-545. doi:10.1126/science.3399889

39. Heaney CA, Israel BA. Social networks and social support. In: Glanz K, Rimer BK, Viswanath K, editors. Health behavior and health education: Theory, research, and practice. Jossey-Bass; 2008. p. 189-210.

40. Klassen AC, Washington C. How does social integration influence breast cancer control among urban African-American women? Results from a cross-sectional survey. BMC Women's Health. 2008 Feb 6;8(1).

41. Jones CJ, Smith H, Llewellyn C. Evaluating the effectiveness of health belief model interventions in improving adherence: A systematic review. Health Psychology Review [Internet]. 2013 Jun 12;8(3):253–69. Available from: https://www.tandfonline.com/doi/abs/10.1080/17437199.2013.802623

42. de Vries H. An Integrated Approach for Understanding Health Behavior; The I-Change Model as an Example. Psychology and Behavioral Science International Journal. 2017 Mar 9;2(2).

43. de Vries H, Mesters I, van de Steeg H, Honing C. The general public's information needs and perceptions regarding hereditary cancer: an application of the Integrated Change Model. Patient Education and Counseling. 2005 Feb;56(2):154–65.

44. Prochaska JO, Velicer WF. The Transtheoretical Model of Health Behavior Change. American Journal of Health Promotion [Internet]. 1997;12(1):38–48. Available from: https://doi.org/10.4278/0890-1171-12.1.38

45. Dellaert BGC, Swait J, Adamowicz WLV, Arentze TA, Bruch EE, Cherchi E, et al. Individuals' Decisions in the Presence of Multiple Goals [Internet]. Customer Needs and

For peer review only

Classification: Internal

Solutions. 2017 May 11;5(1-2):51–64. Available from: https://doi.org/10.1007/s40547-017-0071-1

46. Krueger RA, Casey MA. Focus groups: a practical guide for applied research. 5th ed. Thousand Oaks, California: Sage; 2015.

47. Bryman A. Social Research Methods. 5th ed. London: Oxford University Press; 2015.

48. Creswell J. Qualitative inquiry and research design. Thousand Oaks, Calif.: Sage; 2007.

49. Pachur T, Spaar M. Domain-specific preferences for intuition and deliberation in decision making. J Applied Res Memory Cognition. 2015;4:303-311.

50. Chew LD, Bradley KA, Boyko EJ. Brief questions to identify patients with inadequate health literacy. Fam Med. 2004;36(8):588-594.

51. Antonucci T. Hierarchical mapping technique. Gen J Am Soc. 1986;10:10-2.

52. Strauss AL, Corbin J. Basics of qualitative research: Grounded Theory procedures and techniques. Thousand Oaks: Sage publications; 1990.

53. Linstone HA, Turoff M. The Delphi Method: Techniques and Applications. Technometrics. 1975 Aug;18(3):363.

54. von der Gracht HA. Consensus measurement in Delphi studies. Technological Forecasting and Social Change. 2012 Oct;79(8):1525–36.

For peer review only

Classification: Internal

Standards for Reporting Qualitative Research (SRQR)*

http://www.equator-network.org/reporting-guidelines/srqr/

Page/line no(s).

Title and abstract

**Title** - Concise description of the nature and topic of the study Identifying the study as qualitative or indicating the approach (e.g., ethnography, grounded theory) or data collection methods (e.g., interview, focus group) is recommended	Title page
**Abstract** - Summary of key elements of the study using the abstract format of the intended publication; typically includes background, purpose, methods, results, and conclusions	Abstract (p. 3)

Introduction

**Problem formulation** - Description and significance of the problem/phenomenon studied; review of relevant theory and empirical work; problem statement	Introduction (p. 5)
**Purpose or research question** - Purpose of the study and specific objectives or questions	Objectives (p. 5-6)

Methods

**Qualitative approach and research paradigm** - Qualitative approach (e.g., ethnography, grounded theory, case study, phenomenology, narrative research) and guiding theory if appropriate; identifying the research paradigm (e.g., postpositivist, constructivist/ interpretivist) is also recommended; rationale**	Described on p. 8-12 per section of planned study.
**Researcher characteristics and reflexivity** - Researchers' characteristics that may influence the research, including personal attributes, qualifications/experience, relationship with participants, assumptions, and/or presuppositions; potential or actual interaction between researchers' characteristics and the research questions, approach, methods, results, and/or transferability	Not included in the protocol, but will be included in publications resulting from the planned study.
**Context** - Setting/site and salient contextual factors; rationale**	p. 10 (interviews) and p. 12 (Delphi method)
**Sampling strategy** - How and why research participants, documents, or events were selected; criteria for deciding when no further sampling was necessary (e.g., sampling saturation); rationale**	Planned recruitment strategies for interviews are described on p. 10-11
**Ethical issues pertaining to human subjects** - Documentation of approval by an appropriate ethics review board and participant consent, or explanation for lack thereof; other confidentiality and data security issues	Ethics and Dissemination (p. 13)

For peer review only - http://bmjopen.bmj.com/site/about/guidelines.xhtml

Classification: Internal

**Data collection methods** - Types of data collected; details of data collection procedures including (as appropriate) start and stop dates of data collection and analysis, iterative process, triangulation of sources/methods, and modification of procedures in response to evolving study findings; rationale**	Described in Research Design for each of the three steps (p. 6-12).
**Data collection instruments and technologies** - Description of instruments (e.g., interview guides, questionnaires) and devices (e.g., audio recorders) used for data collection; if/how the instrument(s) changed over the course of the study	Described in Research Design for each of the three steps (p. 6-12).
**Units of study** - Number and relevant characteristics of participants, documents, or events included in the study; level of participation (could be reported in results)	Described in Research Design for each of the three steps (p. 6-12).
**Data processing** - Methods for processing data prior to and during analysis, including transcription, data entry, data management and security, verification of data integrity, data coding, and anonymization/de-identification of excerpts	Described in Research Design for each of the three steps (p. 6-12).
**Data analysis** - Process by which inferences, themes, etc., were identified and developed, including the researchers involved in data analysis; usually references a specific paradigm or approach; rationale**	Described in Research Design for each of the three steps (p. 6-12).
**Techniques to enhance trustworthiness** - Techniques to enhance trustworthiness and credibility of data analysis (e.g., member checking, audit trail, triangulation); rationale**	Described in Research Design for each of the three steps (iterative mixed-method approach; p. 6-12).

Results/findings

**Synthesis and interpretation** - Main findings (e.g., interpretations, inferences, and themes); might include development of a theory or model, or integration with prior research or theory	N/A (outcomes of proposed study are described in Objectives section (p. 5-6))
**Links to empirical data** - Evidence (e.g., quotes, field notes, text excerpts, photographs) to substantiate analytic findings	N/A

Discussion

For peer review only
For peer review only - http://bmjopen.bmj.com/site/about/guidelines.xhtml
Classification: Internal

**Integration with prior work, implications, transferability, and contribution(s) to the field -** Short summary of main findings; explanation of how findings and conclusions connect to, support, elaborate on, or challenge conclusions of earlier scholarship; discussion of scope of application/generalizability; identification of unique contribution(s) to scholarship in a discipline or field	N/A
**Limitations** - Trustworthiness and limitations of findings	N/A

Other

**Conflicts of interest** - Potential sources of influence or perceived influence on study conduct and conclusions; how these were managed	Title page: Competing Statement
**Funding** - Sources of funding and other support; role of funders in data collection, interpretation, and reporting	Title page: Funding Statement

*The authors created the SRQR by searching the literature to identify guidelines, reporting standards, and critical appraisal criteria for qualitative research; reviewing the reference lists of retrieved sources; and contacting experts to gain feedback. The SRQR aims to improve the transparency of all aspects of qualitative research by providing clear standards for reporting qualitative research.

**The rationale should briefly discuss the justification for choosing that theory, approach, method, or technique rather than other options available, the assumptions and limitations implicit in those choices, and how those choices influence study conclusions and transferability. As appropriate, the rationale for several items might be discussed together.

Reference:
O'Brien BC, Harris IB, Beckman TJ, Reed DA, Cook DA. **Standards for reporting qualitative research: a synthesis of recommendations.** *Academic Medicine*, Vol. 89, No. 9 / Sept 2014 DOI: 10.1097/ACM.0000000000000388

For peer review only - http://bmjopen.bmj.com/site/about/guidelines.xhtml

Classification: Internal

BMJ Open

A Socially Interdependent Choice Framework for Social Influences in Healthcare Decision-Making: A Study Protocol

Journal:	*BMJ Open*
Manuscript ID	bmjopen-2023-079768.R2
Article Type:	Protocol
Date Submitted by the Author:	05-Feb-2024

Complete List of Authors:	Nouwens, Sven; Erasmus Universiteit Rotterdam Erasmus School of Health Policy and Management; Erasmus University Rotterdam, Erasmus Choice Modeling Centre<br>Veldwijk, Jorien; Erasmus Universiteit Rotterdam Erasmus School of Health Policy and Management; Erasmus University Rotterdam, Erasmus Choice Modeling Centre<br>Pilli, Luis; Erasmus Universiteit Rotterdam Erasmus School of Health Policy and Management; Erasmus University Rotterdam, Erasmus Choice Modeling Centre<br>Swait, J.D.; Erasmus Universiteit Rotterdam Erasmus School of Health Policy and Management; Erasmus University Rotterdam, Erasmus Choice Modeling Centre<br>Coast, Joanna; University of Bristol, Population Health Sciences<br>de Bekker-Grob, Esther; Erasmus Universiteit Rotterdam Erasmus School of Health Policy and Management; Erasmus University Rotterdam, Erasmus Choice Modeling Centre

**Primary Subject Heading**:	Health economics
Secondary Subject Heading:	Health economics, Health policy
Keywords:	Social Interaction, HEALTH ECONOMICS, Social Support

SCHOLARONE™
Manuscripts

A Socially Interdependent Choice Framework for Social Influences in Healthcare Decision-Making: A Study Protocol

S.P.H. Nouwens[1,2,3*], J. Veldwijk[1,2,3], L. Pilli[1,2,3], J.D. Swait[1,2], J. Coast[4], E.W. de Bekker-Grob[1,2,3]

[1] Erasmus School of Health Policy & Management, Erasmus University Rotterdam

[2] Erasmus Choice Modeling Centre, Erasmus University Rotterdam, the Netherlands

3 Erasmus Centre for Health Economics Rotterdam, Erasmus University, the Netherlands

[4] Health Economics Bristol, Population Health Sciences, Bristol Medical School, University of Bristol, UK

* Correspondence to:

S.P.H. Nouwens

Erasmus School of Health Policy & Management

Erasmus University Rotterdam

P.O. Box 1738

3000 DR Rotterdam

The Netherlands

nouwens@eshpm.eur.nl

For peer review only

Funding Statement

The project has received funding from the Dutch Research Council (NWO-Talent-Scheme-Vidi-Grant No. 09150171910002). Joanna Coast is supported by the Wellcome Trust [205384/Z/16/Z]. For the purpose of open access, the author has applied a CC BY public copyright license to any Author Accepted Manuscript version arising from this submission.

Competing Statement

All authors have completed the ICMJE uniform disclosure form at http://www.icmje.org/disclosure-of-interest/ and declare: no support from any organisation for the submitted work; no financial relationships with any organisations that might have an

Classification: Internal

interest in the submitted work in the previous three years; no other relationships or activities that could appear to have influenced the submitted work.

Word Count: 3532

Keywords: Social Interaction, Social Environment, Decision-Making, Patient Preferences, Choice Behaviour

For peer review only

Classification: Internal

Abstract

Objectives Current choice models in healthcare (and beyond) can provide suboptimal predictions of healthcare users' decisions. One reason for such inaccuracy is that standard microeconomic theory assumes that decisions of healthcare users are made in a social vacuum. Healthcare choices, however, can in fact be (entirely) socially determined. To achieve more accurate choice predictions within healthcare and therefore better policy-decisions, the social influences that affect healthcare user decision-making need to be identified and explicitly integrated into choice models. The purpose of this study is to develop a socially interdependent choice framework of healthcare user decision-making.

Design A mixed methods approach will be used. A systematic literature review will be conducted that identifies the social influences on healthcare user decision-making. Based on the outcomes of a systematic literature review, an interview guide will be developed that assesses which, and how, social influences affect healthcare user decision-making in four different medical fields. This guide will be used during two exploratory focus groups to assess the engagement of participants and clarity of questions and probes. The refined interview guide will be used to conduct the semi-structured interviews with healthcare professionals and users. These interviews will explore in detail which, and how, social influences affect healthcare user decision-making. Focus group and interview transcripts will be analysed iteratively using a constant comparative approach based upon a mix of inductive and deductive coding. Based on the outcomes a social influence independent choice framework for healthcare user decision-making will be drafted. Finally, the Delphi technique will be employed to achieve consensus about the final version of this choice framework.

Ethics and dissemination This study was approved by the Erasmus School of Health Policy & Management Research Ethics Review Committee (ESHPM, Rotterdam, The Netherlands; reference ETH2122-0666).

Strengths and weaknesses of this study

- This is the first study that will attempt to develop a socially interdependent choice framework of healthcare user decision-making.
- Through applying a mixed method approach, existing knowledge and empirical evidence will be combined to ensure a rigorous approach for framework development.

For peer review only

Classification: Internal

- Inclusion of articles based on the study's title and abstract in the systematic literature review step may result in potentially relevant areas remaining unexamined. In part, this will be corrected in the interviews and Delphi method steps, which allow influences and influence mechanisms to surface later in the study.
- Participant recruitment via healthcare providers may lead to selection bias. However, recruited participants will likely be experienced with healthcare decision-making within a medical field of interest.

For peer review only

Classification: Internal

Introduction

Rising expenditures, an ageing population, high prices of new medical treatment options, and substantial annual resource waste put healthcare under heavy pressure (1-4). To avoid poor policy decisions, trial-and-error implementation and demand-supply imbalance, there is a need for accurate predictions of healthcare users' choice behaviour (5, 6). Current choice models in healthcare (and beyond), however, provide inaccurate predictions of individual healthcare user decisions. One reason for such inaccuracy is that standard microeconomic theory assumes that decision-making occurs in a social vacuum. Choice behaviour, under this *homo economicus* perspective (7), is regarded as a completely rational process in which the decision maker is driven purely by their own interest, disregarding any self- or other-originating psychological factors such as social influences. However, healthcare user choices can in fact be (entirely) socially determined (8-12). To achieve more accurate choice behaviour predictions within healthcare and therefore better policy-decisions, the social influences that affect healthcare users' decision-making need to be identified and explicitly integrated into choice models.

This motivates the need for an expanded choice paradigm that must be developed and validated. In the pursuit of this objective, a first important step is to develop and extend the theory of decision-making by building a conceptual framework that depicts which social influences impact healthcare user decision-making, and how they achieve their impact. With this in mind, we developed a systematic approach that 1) empirically identifies these social influences and 2) suggests mechanisms for their impacts. We strive towards these objectives using a phased approach: a systematic literature review, a series of focus groups and semi-structured interviews, and the Delphi method will supplement each other. This paper provides the protocol for the systematic approach that will be taken towards developing this socially interdependent choice framework.

Objectives

This study is part of the INTERSOCIAL project that aims to develop and validate a socially interdependent choice paradigm for healthcare user decision-making such that more accurate predictions of choice behaviour in healthcare can be achieved (13). This study protocol will focus on conducting the necessary steps that will guide the attempt to extend the existing theoretical choice framework to account for social influences. The objectives for this study protocol are:

For peer review only

Classification: Internal

1. to identify which, and how, social influences have previously been found to affect healthcare user decision-making based on existing published peer-reviewed studies;
2. to explore with healthcare users in four different areas which, and how, social influences affect healthcare user decision-making; and
3. to develop consensus among experts (including healthcare professionals) about which, and how, social influences affect healthcare user decision-making.

Definition of Social Influence

There exist many definitions of social influence (14-17) with different levels of practical applicability. Based on a consensus meeting with the research team and external experts (n=16) from different academic fields, it was decided to use the following definition in the present study: "*social influence* is the process by which an individual's behaviour, thoughts, beliefs, attitudes, feelings, actions, preferences, decisions, or choice behaviour are (directly and/or indirectly) affected by other people." These 'other people' can be anyone that the decision maker (in our case, users of healthcare) is in direct contact with but also individuals that the decision maker is not in direct contact with, e.g., celebrity endorsements (18).

Note that our definition is centred on a decision maker whose healthcare decisions may depend upon other people in the decision maker's social circle, implying that we are excluding the study of networks of individual healthcare users who are jointly influenced by each other (19). We apply a unidirectional paradigm where the entire social network is centred on a single healthcare user and study how this social network ((types of) individuals and processes) influences the healthcare user, but not how the healthcare user influences others' social networks or how a healthcare user's social contacts are influenced by each other. We only include social influences that can be tracked to a single source. Other social influences (such as culture (20), education (21) and perceived discrimination (22)) that cannot be tracked to a single source will be documented only.

Research Design

The research process will use a mixed-methods approach. First, a systematic literature review that identifies the (types of) individuals and the processes that systematically influence healthcare users' decisions within different medical fields will be performed. The results from the systematic literature review will aid in constructing an interview guide for the subsequent round of (group and individual) interviews. As individuals are often unconscious and unaware

For peer review only

Classification: Internal

of the degree to which they are socially influenced (23-24), the results from the systematic literature review can additionally help to construct interview questions that stimulate recall and awareness of social influences that might otherwise not be raised by respondents. Second, two focus groups and forty semi-structured interviews will be conducted to explore and assess healthcare users' and healthcare professionals' views on social influences on healthcare user decision-making. The focus groups have a secondary aim of testing engagement of participants with the topic and assessing the clarity of questions and probes in the interview guide. Their feedback will then be used to refine the interview guide for the semi-structured interviews. Third, the Delphi method will be applied to reach consensus among experts (including healthcare professionals and choice modellers) about the socially interdependent choice framework on healthcare user decision-making.

For the systematic literature review the following medical fields of study have been chosen: vaccination, birth setting, arthroplasty, and prostate cancer treatment. The rationale for choosing these fields is fourfold. First, healthcare users in these fields face a degree of autonomy in their choice, meaning that they are not obliged to choose one alternative over the other, which is essential for testing real choice behaviour predictability. Second, many healthcare users face these medical fields (in the Netherlands, which has 17.5 million inhabitants: almost all people are affected by vaccinations, between 160,000 and 180,000 people give birth yearly (25), 31,514 hip replacements were reported in 2022 (26), and prevalence of prostate cancer is over 55,000 (27)) speaking to relevance from the perspective of development of an extended socially interdependent framework. Third, a higher degree of generalizability is achieved by including medical fields that are likely to differ in types/degrees of social interdependence and consequences that may employ different mechanisms of social influence. Fourth, in each of these four fields, there are current policy challenges related to the decisions made by healthcare users in these areas: i) the demand for influenza vaccination is lower than for other vaccinations (28), ii) there is increasing interest in birth centres in the Dutch population (29), iii) there is an exponential rise in patients with joint problems (30-31), and iv) there is an alarming increase in active-surveillance drop-out from prostate cancer patients (32-33).

Systematic Literature Review

Initially, a systematic literature review of social influences on healthcare user decision-making will be performed. The review will follow PRISMA guidelines (34) and map the

For peer review only

Classification: Internal

literature in three dimensions that concern the development of the socially interdependent choice paradigm proposed by the INTERSOCIAL project (13). These dimensions describe i) the individuals or groups influencing health-related decision-making; ii) the mechanisms through which they influence decisions of healthcare users; and iii) the choice constructs that these processes affect. A narrative synthesis of the sources and mechanisms of social influence and choice constructs affected will be provided.

Concerning the first dimension, psychosocial variables are one of the key facets of patient-centred care (35). The past literature offers evidence of treatment choices as an action embedded in a network of social relationships, including experts and non-experts (36-37). This first dimension focuses on which individuals' and groups' actions must be considered for the development of the theoretical framework for healthcare user decision-making.

The second dimension, i.e., social influence mechanisms, refers to the processes by which a healthcare user's behaviour, thoughts, beliefs, attitudes, feelings, actions, preferences, decisions, or choice behaviours are (directly and/or indirectly) affected by other people (38-39). Mechanisms and dimensions already present in the health-related literature will support classifying included papers into categories. Physician-patient interaction (36-37) and social support (40) are two relevant and well-defined constructs that may impact healthcare user decision-making. Social norm is a relevant construct from socio-cognitive theories, and it is present in many attitudinal-behavioural models in the health domain, such as the Health Belief (41) model, I-Change (42-43) model, and the Transtheoretical Model of Behavior Change (44).

The creation of a socially interdependent choice framework demands the connection between the first two dimensions and the choice constructs involved in choice econometric models, such as the choice framework of Dellaert et al. (45), which describes how individuals evaluate attributes and make decisions in the presence of multiple goals. Since insights about this connection from the extant literature are sparse at best, choice constructs will be aggregated into three broadly defined main categories. These categories contain the effects of social influences on (1) healthcare users' goals, (2) preference formation, and (3) non-evaluative components of choice models. Goals involve crucial personal trade-offs such as life expectancy versus quality of life, where different alternatives are valued differently based on the goal that a healthcare user seeks to attain, with other persons possibly influencing the activation of certain goals. Preference formation describes the effects of social influences on

For peer review only - http://bmjopen.bmj.com/site/about/guidelines.xhtml

Classification: Internal

the acquisition of information about attributes and the deployment of weights to these attributes to determine options' total utilities. Finally, non-evaluative components refer to any steps of the decision-making process where certain options are excluded based on single attributes, instead of an option's unique blend of attribute levels. Examples of non-evaluative components are habitual behaviour, non-compensatory processes, or screening of alternatives before evaluation.

To attain the objectives of the systematic literature review, nine different databases will be searched: Embase, Cochrane CENTRAL, Econlit, Google Scholar, International Bibliography of the Social Sciences, Medline, Psychinfo, Scopus, and Web of Science. To be eligible, a paper must return from a search containing (variations around) the keywords "social influence" AND "decision-making" AND "empirical data" AND "healthcare field". The "healthcare field" differs for each of the four healthcare fields studied. For example, the field of vaccination will use the keywords "vaccine" OR "vaccine hesitancy" OR "vaccination" OR "vaccination coverage". We will exclude the terms "parental" and "parental decision-making", as we are interested in how a healthcare user makes choices about their own health and how social influences affect these choices.

The inclusion criteria further require that the full text of a paper is accessible through the institutional subscription of any of the project collaborators, is written in English, and presents results of original empirical data collected in Western countries. Countries that will be included in the search strategy are the United States, Canada, North America, United Kingdom, England, Ireland, Northern Ireland, Scotland, The Netherlands, Belgium, France, Germany, Denmark, Norway, Sweden, Finland, Spain, Portugal, Italy, Austria, Switzerland, Iceland, Luxembourg, Liechtenstein, San Marino, Monaco, Andorra, Malta, Cyprus, Gibraltar, Europe, Australia, and New Zealand. Papers reporting data from other countries will be excluded due to the assumption of significantly divergent cultures and social environments compared to Western countries.

The analysis will follow a two-step deductive-inductive approach. First, in the deductive step, we will extract data from papers coding the social influence sources, mechanisms (such as doctor-patient interaction, or social support), and choice constructs using the framework from Dellaert et al. (45). From this initial data extraction round, we will streamline the definitions of social influence mechanisms and choice constructs. Based on the streamlined definitions, we will inductively propose the connection of sources and mechanisms of social influence and

For peer review only

Classification: Internal

choice constructs. The last step of the process is to describe frequencies and co-occurrences of relevant individuals and groups, social influence mechanisms and choice constructs, and differences across medical areas.

From a broader perspective, the systematic literature review will support the identification of important social influences, their mechanisms, and affected choice constructs in the extant literature. This will allow the subsequent steps to exploit the signals and explore the gaps.

Exploratory Focus Groups and Semi-Structured Interviews

Based on the outcomes of the systematic literature review, using best-practice guidelines (46, 47), an interview guide will be developed that assesses which, and how, social influences affect healthcare user decision-making according to healthcare users. Two exploratory focus groups will be conducted to enable the further development of the content within the interview guide through discussion of issues that the research team may not have considered, to test the engagement of participants with the topic, and to assess the clarity of questions and probes in the interview guide (and make any adjustments). One focus group will be conducted with people aged 60 years and older, who were or will be offered flu vaccination and the second focus group among pregnant women who were or are choosing their birth setting. Each group will consist of 6-10 participants (46) and will be conducted in-person.

One important aspect of the focus groups is to test and further generate the interview guide. As this is a complex topic which may not be immediately understandable to participants, attention will be given to whether questions were understood by the participants (i.e., not considered too abstract in nature), whether the formulation of the questions stimulated understanding of the topic, and whether probes were clear. The focus groups may also introduce topics that were not expected, and the interview guide will be adjusted to enable these topics to be captured during individual interviews.

Subsequently, semi-structured interviews will be conducted. The semi-structured interviews will provide a detailed exploration of which, and how, social influences affect healthcare user decision-making. These interviews will be conducted among healthcare users (decision makers) and healthcare professionals in the medical fields of influenza vaccination, prostate cancer treatment, joint replacement, and birth setting. Per medical field, a minimum of 10 individuals will be recruited consisting of healthcare users (n=5) and healthcare professionals (n=5) (48). Additional interviews will be added based on whether data saturation has been

For peer review only

Classification: Internal

reached within each medical field of interest. Interview guides will be adjusted as the analysis progresses, to take account of new and emerging findings and to pursue new lines of enquiry. Interviews will preferably be face-to-face at a convenient location for the interviewee, however if requested by the interviewee, these in-depth interviews can be conducted either via a networking facility (e.g., Microsoft Teams or Zoom) or telephone.

Participants will be recruited through already confirmed collaborations with Erasmus MC – University Medical Centre clinicians, general practices, obstetric centre (focus groups and interviews), and the Prostate Centre South-West Netherlands (interviews only). Focus group participants will receive a €20 gift voucher as compensation. For the interviews, participants will receive a €10 gift voucher as compensation if they participate in a face-to-face or online interview. Focus groups and interviews will be audio-recorded, then transcribed verbatim.

Before the focus groups and in-person interviews begin, participants will be provided with information about the study, and asked to sign an informed consent form. After consent has been obtained, they will be asked to fill in a short (online) survey including questions on their health literacy (49) and their decision-making style (50). At the end of the interviews, participants will be presented with their survey answers and given the opportunity to revise them if they would like to do so in light of the discussion.

As part of the interviews, hierarchical mapping will be employed to construct a social proximity map (51). Participants will be asked to place influential individuals on a diagram of concentric circles, with the decision maker (i.e., healthcare user) in the middle. The placement of individuals represents the participant's social network, but also the strength of influence of each specific individual.

The analysis of the transcripts from the focus groups will be conducted prior to the interviews and will focus on the issues around the data collection process, as well as providing some preliminary themes. Transcripts from interviews will be analysed iteratively using a grounded theory constant comparative approach (52) based upon a mix of inductive and deductive (from the systematic review/initial framework) coding. The constant comparative approach compares each incident in the data with other incidents for similarities and differences and groups conceptual similarities under higher-level descriptive concepts (52). The analysis will result in a first version of the theoretical framework of social influence on healthcare user decision-making. For data analysis Atlas.ti will be used. Data collected through survey and

For peer review only

Classification: Internal

recording devices will be stored for at least ten years through SURF Yoda, in line with Erasmus University policy.

Delphi Method

The findings obtained in the literature review and qualitative interviews will be consolidated by applying the Delphi method among experts from different medical (influenza vaccination, prostate cancer treatment, joint replacement and birth setting) and scientific (e.g., choice modelling in health economics, marketing, transportation, environmental economics) fields. Linstone & Turoff (53) introduced the Delphi method as "*a method for structuring a group communication process so that the process is effective in allowing a group of individuals, as a whole, to deal with a complex problem.*" The current study will utilize this method to further structure and reach consensus on the developed socially interdependent choice framework.

Experts (researchers and clinicians) from the different fields will be proposed parts of the socially interdependent choice framework and asked to provide feedback in several rounds. Experts will be contacted through email and are given a week to reply to a round of the Delphi method. After each round, a summary of the experts' perspectives will be provided to the experts, along with a revised model of social influence. This will allow the experts to revise their earlier comments in the next round of the Delphi study.

Agreement among two-thirds (67%) of participants on a model of social influence will be considered the threshold for consensus (53). Besides consensus, group stability will also be measured. This gives an indication of consistency across rounds. A set of Chi square tests will be performed to keep track of group stability (54). If stability and consensus have both been reached, the procedure will be stopped. If after five rounds, stability or consensus has not been reached, the procedure will also be stopped. In this case, the version of the socially interdependent framework with the most consensus will be accepted, and dissents will be noted.

Patient Involvement

Healthcare users and members of the public were not involved in the design, conduct, reporting, or dissemination of this protocol. However, experiences of healthcare users will guide the refinement of the interview guide and eventually the creation of a socially interdependent choice framework in the planned study.

For peer review only

Classification: Internal

Discussion

This study will focus on identifying which social influences affect healthcare user decision-making, as well as the mechanisms/processes for their impacts. By forming an understanding of social influences in individual decision-making, this study will not only help to identify social influences in healthcare decision-making but also help to extend choice theory by building an evidence-based interdependent choice framework that depicts which, and how, social influences impact healthcare user decision-making. By incorporating these social influences, which can have a big impact on the choice process (8-12), such a framework has the potential to contribute towards more accurate predictions of healthcare choices, which could in turn have a positive impact on decreasing poor policy decisions, trial-and-error implementations, and demand-supply imbalances.

Addressing limitations

A limitation of the systematic literature review approach is the inclusion criteria of articles based on the study's title and abstract. This may result in missing findings regarding less straightforward mechanisms of social influences or choice constructs, that are not covered by the search strategy. Additionally, minor findings are more likely to be overlooked, as these are often not mentioned in the abstract or title. This may lead to an overrepresentation of significant results resulting from the literature review. Further validation would be necessary to estimate the effects and their significance more accurately. The current protocol is designed to (partly) correct for this: interviews and the Delphi method allow influences and influence mechanisms that are not found in the systematic literature step to surface later in the study.

Another limitation, related to the explorative focus groups and semi-structured interviews, is the recruitment of participants through healthcare providers. While on the one hand this can count as a strength due to recruiting participants who are likely experienced with healthcare decision-making within a medical field of interest, it may also lead to selection bias: these participants may not be representative of the targeted population in terms of experience with the decisions that are studied.

Ethics and Dissemination

This study was approved by the Erasmus School of Health Policy & Management Research Ethics Review Committee (ESHPM, Rotterdam, The Netherlands; reference ETH2122-0666).

For peer review only - http://bmjopen.bmj.com/site/about/guidelines.xhtml

Classification: Internal

All participants will be provided with an information letter and given the opportunity to ask questions on the study. Informed consent will be obtained before their participation. Data will be anonymised by removing names and any other personal information from transcripts and answers during the Delphi method.

Findings will be published in peer-reviewed academic journals and further shared with various audiences such as researchers, healthcare professionals and policy makers through methods such as public presentations and academic conferences. The gathered data and the constructed data set from the systematic literature review can be requested from the corresponding author. The full interview transcripts will not be made available for request as participants have signed a consent form that promises to maintain complete privacy.

Acknowledgement

The authors wish to thank Angie Fagerlin for fruitful discussion and comments on an earlier version.

Authors' Contribution

EWBG, JV, JDS, LP, JC: initial conceptualization and design of the study. All authors further adapted and improved the design of the study. SPHN: drafted the manuscript. All authors provided critical revision of the manuscript and have read and approved the final manuscript.

For peer review only

Classification: Internal

References

1. West LM, Diack L, Cordina M, Stewart D. A systematic review of the literature on "medication wastage": an exploration of causative factors and effect of interventions. International Journal of Clinical Pharmacy. 2014 Jul 19;36(5):873–81.

2. Bloom DE, Chatterji S, Kowal P, Lloyd-Sherlock P, McKee M, Rechel B, et al. Macroeconomic implications of population ageing and selected policy responses. The Lancet [Internet]. 2015 Feb [cited 2023 Aug 4];385(9968):649–57. Available from: https://www.ncbi.nlm.nih.gov/pmc/articles/PMC4469267/

3. OECD, European Union. Health at a Glance: Europe 2018: state of health in the EU cycle. [Internet]. Brussels: OECD Publishing; 2018 [cited 2023 Jul 21]. Available from: https://doi.org/10.1787/health_glance_eur-2018-en.

4. Watkins JB. Affordability of health care: a global crisis. Value Heal. 2018;21(3):280-282.

5. Zucchelli E, Jones A, Rice N. The evaluation of health policies through microsimulation methods. Int J of Microsim, 2012;5(1):2-20.

6. Terris-Prestholt F, Quaife M, Vickerman P. Parameterising User Uptake in Economic Evaluations: The role of discrete choice experiments. Health Economics. 2016 Jan 15;25(1):116–23.

7. McFadden D. Free markets and fettered consumers. American Economic Review. 2006 Feb;96(1):5–29.

8. Hopfer S, Clippard JR. College Women's HPV Vaccine Decision Narratives. Qualitative Health Research. 2010 Sep 14;21(2):262–77.

9. Marsh HA, Malik F, Shapiro E, Omer SB, Frew PM. Message framing strategies to increase influenza immunization uptake among pregnant African American women. Maternal and Child Health Journal. 2013 Dec 12;18(7):1639–47.

For peer review only

Classification: Internal

10. Edge R, Goodwin D, Isba R, Keegan T. Socialization, indifference, and convenience: exploring the uptake of influenza vaccine among medical students and early career doctors. Qualitative Health Research. 2017 Jul 24;27(13):1982–93.

11. Lewis S, Kenny K, Broom A, Kirby E, Boyle F. The social meanings of choice in living-with advanced breast cancer. Social Science & Medicine. 2021 Jul;280:114047.

12. de Bekker-Grob EW, Howard K, Swait J. Identifying the impact of social influences in health-related discrete choice experiments [Internet]. PLoS ONE. 2022 [cited 2023 Jul 21];17(10):e0276141. Available from: https://doi.org/10.1371/journal.pone.0276141

13. de Bekker-Grob EW, Donkers B, Bliemer M, Coast J, Swait J. Towards Accurate Prediction of Healthcare Choices: The INTERSOCIAL Project. The Patient [Internet]. 2022 Sep 1 [cited 2023 Aug 5];15(5):509–12. Available from: https://pubmed.ncbi.nlm.nih.gov/35881234/

14, Abrams D, Hogg MA. Social identification, self-categorization and social influence. European Review of Social Psychology. 1990 Jan;1(1):195–228.

15. Turner JC. Social influence. Milton Keynes: Open Univ. Press; 1991.

16. Leenders R. Modeling social influence through network autocorrelation: constructing the weight matrix. Social Networks. 2002 Jan;24(1):21–47.

17. Gass RH. Sociology of social influence. In: Wright JD, editor. International encyclopedia of the social and behavioral sciences (2nd ed). Oxford: Elsevier; 2015. p. 348–54.

18. Troiano G, Nante N, Cozzolino M. The Angelina Jolie effect – Impact on breast and ovarian cancer prevention: A systematic review of effects after the public announcement in May 2013. Health Education Journal. 2017 Jun 24;76(6):707–15.

19. Otte E, Rousseau R. Social network analysis: a powerful strategy, also for the information sciences. Journal of Information Science. 2002 Dec;28(6):441–53.

For peer review only

Classification: Internal

20. Lee SY. Cultural factors associated with breast and cervical cancer screening in Korean American women in the US: an integrative literature review. Asian Nursing Research. 2015 Jun;9(2):81–90.

21. Damiani G, Basso D, Acampora A, Bianchi CBNA, Silvestrini G, Frisicale EM, et al. The impact of level of education on adherence to breast and cervical cancer screening: Evidence from a systematic review and meta-analysis. Preventive Medicine. 2015 Dec;81:281–289.

22. Jacobs EA, Rathouz PJ, Karavolos K, Everson-Rose SA, Janssen I, Kravitz HM, et al. Perceived Discrimination Is Associated with Reduced Breast and Cervical Cancer Screening: The Study of Women's Health Across the Nation (SWAN). Journal of Women's Health. 2014 Feb;23(2):138–145.

23. Nolan JM, Schultz PW, Cialdini RB, Goldstein NJ, Griskevicius V. Normative social influence is underdetected. Personality and Social Psychology Bulletin. 2008 May 9;34(7):913–23.

24. Prabhakaran R, Gray JR. The pervasive nature of unconscious social information processing in executive control. Frontiers in Human Neuroscience. 2012;6.

25. Nederlands Jeugdinstituut. Cijfers over geboorte [Internet]. 2023 June 2 [cited 2023 Aug 4]. Available from: https://www.nji.nl/cijfers/geboorte

26. LROI. LROI 2022 [Internet]. LROI. 2022 [cited 2023 Aug 4]. Available from: https://www.lroi.nl/publicaties/jaarrapportage/

27. NKR. NKR Cijfers [Internet]. [cited 2023, Aug 8]. Available from: https://iknl.nl/nkr-cijfers

28. van Lier EA, Hament JM, Knijff M, Westra M, Ernst A, Giesbers H, et al. Vaccination coverage and annual report of the National Immunisation Programme in the Netherlands, 2022. National Institute for Public Health and the Environment. 2022 [cited 2023 Aug 4]. Available from: https://rivm.openrepository.com/handle/10029/626797

For peer review only

Classification: Internal

29. Hermus MAA, Boesveld IC, Hitzert M, Franx A, de Graaf JP, Steegers EAP, et al. Defining and describing birth centres in the Netherlands - a component study of the Dutch Birth Centre Study [Internet]. BMC Pregnancy and Childbirth. 2017 Jul 3 [cited 2023 Jul 21];17(1). Available from: https://doi.org/10.1186/s12884-017-1375-8

30. Woolf AD, Pfleger B. Burden of major musculoskeletal conditions. Bull World Health Organ. 2003;81(9):646-656.

31. Takeda R, Matsumoto T, Yuji Maenohara, Yasunori Omata, Hiroshi Inui, Nagase Y, et al. Increasing trend of radiographic features of knee osteoarthritis in rheumatoid arthritis patients before total knee arthroplasty [Internet]. Scientific Reports. 2022 Jun 21 [cited 2023 Jul 21];12(1):10452. Available from: https://doi.org/10.1038/s41598-022-14440-2

32. Bokhorst LP, Alberts AR, Rannikko A, Valdagni R, Pickles T, Kakehi Y, et al. Compliance Rates with the Prostate Cancer Research International Active Surveillance (PRIAS) Protocol and Disease Reclassification in Noncompliers. European Urology. 2015 Nov;68(5):814–21.

33. Kinsella N, Stattin P, Cahill D, Brown C, Bill-Axelson A, Bratt O, et al. Factors Influencing Men's Choice of and Adherence to Active Surveillance for Low-risk Prostate Cancer: A Mixed-method Systematic Review. Eur Urol. 2018;74(3):261-280. doi:10.1016/j.eururo.2018.02.026

34. Page MJ, McKenzie JE, Bossuyt PM, Boutron I, Hoffmann TC, Mulrow CD, et al. The PRISMA 2020 statement: An updated guideline for reporting systematic reviews. British Medical Journal. 2021 Mar 29;372(71).

35. Shaller D. Patient-Centered Care: What Does It Take? Commonw Fund, 2007;68.

36. Kreling B, Figueiredo MI, Sheppard VL, Mandelblatt JS. A qualitative study of factors affecting chemotherapy use in older women with breast cancer: barriers, promoters, and implications for intervention [Internet]. Psycho-Oncology. 2006 Apr 6 [cited 2023 Jul 21];15(12):1065–76. Available from: https://doi.org/10.1002/pon.1042

For peer review only

Classification: Internal

37. Street RL. Information-giving in medical consultations: The influence of patients' communicative styles and personal characteristics. Social Science & Medicine. 1991 Jan;32(5):541–8.

38. House JS, Landis KR, Umberson D. Social relationships and health. Science. 1988;241(4865):540-545. doi:10.1126/science.3399889

39. Heaney CA, Israel BA. Social networks and social support. In: Glanz K, Rimer BK, Viswanath K, editors. Health behavior and health education: Theory, research, and practice. Jossey-Bass; 2008. p. 189-210.

40. Klassen AC, Washington C. How does social integration influence breast cancer control among urban African-American women? Results from a cross-sectional survey. BMC Women's Health. 2008 Feb 6;8(1).

41. Jones CJ, Smith H, Llewellyn C. Evaluating the effectiveness of health belief model interventions in improving adherence: A systematic review. Health Psychology Review [Internet]. 2013 Jun 12;8(3):253–69. Available from: https://www.tandfonline.com/doi/abs/10.1080/17437199.2013.802623

42. de Vries H. An Integrated Approach for Understanding Health Behavior; The I-Change Model as an Example. Psychology and Behavioral Science International Journal. 2017 Mar 9;2(2).

43. de Vries H, Mesters I, van de Steeg H, Honing C. The general public's information needs and perceptions regarding hereditary cancer: an application of the Integrated Change Model. Patient Education and Counseling. 2005 Feb;56(2):154–65.

44. Prochaska JO, Velicer WF. The Transtheoretical Model of Health Behavior Change. American Journal of Health Promotion [Internet]. 1997;12(1):38–48. Available from: https://doi.org/10.4278/0890-1171-12.1.38

45. Dellaert BGC, Swait J, Adamowicz WLV, Arentze TA, Bruch EE, Cherchi E, et al. Individuals' Decisions in the Presence of Multiple Goals [Internet]. Customer Needs and

For peer review only

Classification: Internal

Solutions. 2017 May 11;5(1-2):51–64. Available from: https://doi.org/10.1007/s40547-017-0071-1

46. Krueger RA, Casey MA. Focus groups: a practical guide for applied research. 5th ed. Thousand Oaks, California: Sage; 2015.

47. Bryman A. Social Research Methods. 5th ed. London: Oxford University Press; 2015.

48. Creswell J. Qualitative inquiry and research design. Thousand Oaks, Calif.: Sage; 2007.

49. Chew LD, Bradley KA, Boyko EJ. Brief questions to identify patients with inadequate health literacy. Fam Med. 2004;36(8):588-594.

50. Pachur T, Spaar M. Domain-specific preferences for intuition and deliberation in decision making. J Applied Res Memory Cognition. 2015;4:303-311.

51. Antonucci T. Hierarchical mapping technique. Gen J Am Soc. 1986;10:10-2.

52. Strauss AL, Corbin J. Basics of qualitative research: Grounded Theory procedures and techniques. Thousand Oaks: Sage publications; 1990.

53. Linstone HA, Turoff M. The Delphi Method: Techniques and Applications. Technometrics. 1975 Aug;18(3):363.

54. von der Gracht HA. Consensus measurement in Delphi studies. Technological Forecasting and Social Change. 2012 Oct;79(8):1525–36.

For peer review only

Classification: Internal

Standards for Reporting Qualitative Research (SRQR)*

http://www.equator-network.org/reporting-guidelines/srqr/

Title and abstract

	Page/line no(s).
**Title** - Concise description of the nature and topic of the study Identifying the study as qualitative or indicating the approach (e.g., ethnography, grounded theory) or data collection methods (e.g., interview, focus group) is recommended	Title page
**Abstract** - Summary of key elements of the study using the abstract format of the intended publication; typically includes background, purpose, methods, results, and conclusions	Abstract (p. 3)

Introduction

**Problem formulation** - Description and significance of the problem/phenomenon studied; review of relevant theory and empirical work; problem statement	Introduction (p. 5)
**Purpose or research question** - Purpose of the study and specific objectives or questions	Objectives (p. 5-6)

Methods

**Qualitative approach and research paradigm** - Qualitative approach (e.g., ethnography, grounded theory, case study, phenomenology, narrative research) and guiding theory if appropriate; identifying the research paradigm (e.g., postpositivist, constructivist/ interpretivist) is also recommended; rationale**	Described on p. 8-12 per section of planned study.
**Researcher characteristics and reflexivity** - Researchers' characteristics that may influence the research, including personal attributes, qualifications/experience, relationship with participants, assumptions, and/or presuppositions; potential or actual interaction between researchers' characteristics and the research questions, approach, methods, results, and/or transferability	Not included in the protocol, but will be included in publications resulting from the planned study.
**Context** - Setting/site and salient contextual factors; rationale**	p. 10 (interviews) and p. 12 (Delphi method)
**Sampling strategy** - How and why research participants, documents, or events were selected; criteria for deciding when no further sampling was necessary (e.g., sampling saturation); rationale**	Planned recruitment strategies for interviews are described on p. 10-11
**Ethical issues pertaining to human subjects** - Documentation of approval by an appropriate ethics review board and participant consent, or explanation for lack thereof; other confidentiality and data security issues	Ethics and Dissemination (p. 13)

For peer review only - http://bmjopen.bmj.com/site/about/guidelines.xhtml

Classification: Internal

**Data collection methods** - Types of data collected; details of data collection procedures including (as appropriate) start and stop dates of data collection and analysis, iterative process, triangulation of sources/methods, and modification of procedures in response to evolving study findings; rationale**	Described in Research Design for each of the three steps (p. 6-12).
**Data collection instruments and technologies** - Description of instruments (e.g., interview guides, questionnaires) and devices (e.g., audio recorders) used for data collection; if/how the instrument(s) changed over the course of the study	Described in Research Design for each of the three steps (p. 6-12).
**Units of study** - Number and relevant characteristics of participants, documents, or events included in the study; level of participation (could be reported in results)	Described in Research Design for each of the three steps (p. 6-12).
**Data processing** - Methods for processing data prior to and during analysis, including transcription, data entry, data management and security, verification of data integrity, data coding, and anonymization/de-identification of excerpts	Described in Research Design for each of the three steps (p. 6-12).
**Data analysis** - Process by which inferences, themes, etc., were identified and developed, including the researchers involved in data analysis; usually references a specific paradigm or approach; rationale**	Described in Research Design for each of the three steps (p. 6-12).
**Techniques to enhance trustworthiness** - Techniques to enhance trustworthiness and credibility of data analysis (e.g., member checking, audit trail, triangulation); rationale**	Described in Research Design for each of the three steps (iterative mixed-method approach; p. 6-12).

Results/findings

**Synthesis and interpretation** - Main findings (e.g., interpretations, inferences, and themes); might include development of a theory or model, or integration with prior research or theory	N/A (outcomes of proposed study are described in Objectives section (p. 5-6))
**Links to empirical data** - Evidence (e.g., quotes, field notes, text excerpts, photographs) to substantiate analytic findings	N/A

Discussion

For peer review only - http://bmjopen.bmj.com/site/about/guidelines.xhtml
Classification: Internal

**Integration with prior work, implications, transferability, and contribution(s) to the field -** Short summary of main findings; explanation of how findings and conclusions connect to, support, elaborate on, or challenge conclusions of earlier scholarship; discussion of scope of application/generalizability; identification of unique contribution(s) to scholarship in a discipline or field	N/A
**Limitations** - Trustworthiness and limitations of findings	N/A

Other

**Conflicts of interest** - Potential sources of influence or perceived influence on study conduct and conclusions; how these were managed	Title page: Competing Statement
**Funding** - Sources of funding and other support; role of funders in data collection, interpretation, and reporting	Title page: Funding Statement

*The authors created the SRQR by searching the literature to identify guidelines, reporting standards, and critical appraisal criteria for qualitative research; reviewing the reference lists of retrieved sources; and contacting experts to gain feedback. The SRQR aims to improve the transparency of all aspects of qualitative research by providing clear standards for reporting qualitative research.

**The rationale should briefly discuss the justification for choosing that theory, approach, method, or technique rather than other options available, the assumptions and limitations implicit in those choices, and how those choices influence study conclusions and transferability. As appropriate, the rationale for several items might be discussed together.

Reference:
O'Brien BC, Harris IB, Beckman TJ, Reed DA, Cook DA. **Standards for reporting qualitative research: a synthesis of recommendations.** *Academic Medicine*, Vol. 89, No. 9 / Sept 2014 DOI: 10.1097/ACM.0000000000000388

For peer review only - http://bmjopen.bmj.com/site/about/guidelines.xhtml
Classification: Internal
